# The parieto-occipital cortex is a candidate neural substrate for the human ability to approximate Bayesian inference

**DOI:** 10.1038/s42003-024-05821-6

**Published:** 2024-02-09

**Authors:** Nicholas M. Singletary, Jacqueline Gottlieb, Guillermo Horga

**Affiliations:** 1 Doctoral Program in Neurobiology and Behavior, Columbia University, New York, NY USA; 2 Department of Neuroscience, Columbia University, New York, NY USA; 3 Zuckerman Mind Brain Behavior Institute, Columbia University, New York, NY USA; 4 New York State Psychiatric Institute, New York, NY USA; 5 Kavli Institute for Brain Science, Columbia University, New York, NY USA; 6 Department of Psychiatry, Columbia University, New York, NY USA

**Keywords:** Decision, Human behaviour

## Abstract

Adaptive decision-making often requires one to infer unobservable states based on incomplete information. Bayesian logic prescribes that individuals should do so by estimating the posterior probability by integrating the prior probability with new information, but the neural basis of this integration is incompletely understood. We record fMRI during a task in which participants infer the posterior probability of a hidden state while we independently modulate the prior probability and likelihood of evidence regarding the state; the task incentivizes participants to make accurate inferences and dissociates expected value from posterior probability. Here we show that activation in a region of left parieto-occipital cortex independently tracks the subjective posterior probability, combining its subcomponents of prior probability and evidence likelihood, and reflecting the individual participants’ systematic deviations from objective probabilities. The parieto-occipital cortex is thus a candidate neural substrate for humans’ ability to approximate Bayesian inference by integrating prior beliefs with new information.

## Abstract (web summary)

Analysis of fMRI data from participants in a probability estimation task suggests that the parieto-occipital cortex is a neural substrate for humans’ ability to approximate Bayesian inference by integrating prior beliefs with new information.

## Introduction

Making adaptive decisions often requires us to infer unobservable, or hidden, states based on probabilistic information. For example, when making a diagnosis, a physician infers an underlying illness based on observable symptoms that provide imperfect evidence for the illness. Furthermore, while probabilistic information can be learned by trial and error, in many situations, inferences rely primarily on described information—such as when the physician relies on reports of the probability of a specific disease or the reliability of a diagnostic test. Probabilistic inference supports a variety of adaptive behaviors in humans and other animals, and alterations in probabilistic inference have been linked to psychopathology^1–4^, underscoring the importance of understanding its neural mechanisms.

According to Bayesian logic, optimal probabilistic inference requires individuals to estimate the posterior probability of a hypothesis by integrating two quantities: the prior probability of the hypothesis and the likelihood of new information conditional on this hypothesis. Although abundant evidence shows that people approximate posterior probabilities consistent with Bayesian principles^5–13^, major questions remain about the mechanisms by which they do so.

A central open question concerns the neural mechanisms supporting not only the encoding, but the integration of prior and likelihood. Previous imaging studies have examined processes that imply Bayesian inference, like change-point detection^14^, information demand^15^, and the neural representations of prior and likelihood uncertainty^16^, but did not ask participants to report the posterior probabilities or examine how the neural representations of these probabilities depend on the prior and likelihood. Other studies did elicit probability estimates, but parametrically manipulated only the prior probability^17^ or only the likelihood^18–22^ while holding the other quantity constant; this practice confounds the posterior probability with the (single) manipulated quantity, eschewing the question of prior–likelihood integration. Finally, Ting et al. ^11^ did independently manipulate prior probability and likelihood, but required participants to choose the option that had higher probability of reward, confounding the integration of these quantities with a representation of expected value (EV).

In the present investigation, we examined this question using fMRI in conjunction with a behavioral task that we developed in which we independently manipulated the prior probability of a hidden state and the likelihood of the evidence conditional on the state. We used a one-shot probability estimation design based on described (numerical) probabilities building on a large behavioral economics literature^5–7,9,13^ showing that this behavior is well captured by Bayesian inference models, avoids complex sequential processes, and reflects many real-life judgments (e.g., from financial investments to legal verdicts). We required participants to estimate the posterior probability of a state based on the prior probability and likelihood, and we used a well-validated incentive-compatible scoring rule^23^ to incentivize accurate estimates and decorrelate posterior probability from EV. We show that a cluster of BOLD activation encompassing the left posterior parietal and anterolateral occipital cortices tracked subjective posterior probability and its subcomponents of prior probability and evidence likelihood and, moreover, correlated with inter-individual variability in estimation strategies, identifying this area as a candidate neural substrate for Bayesian integration.

## Results

### Museum Inference Task

**Fig. 1:**
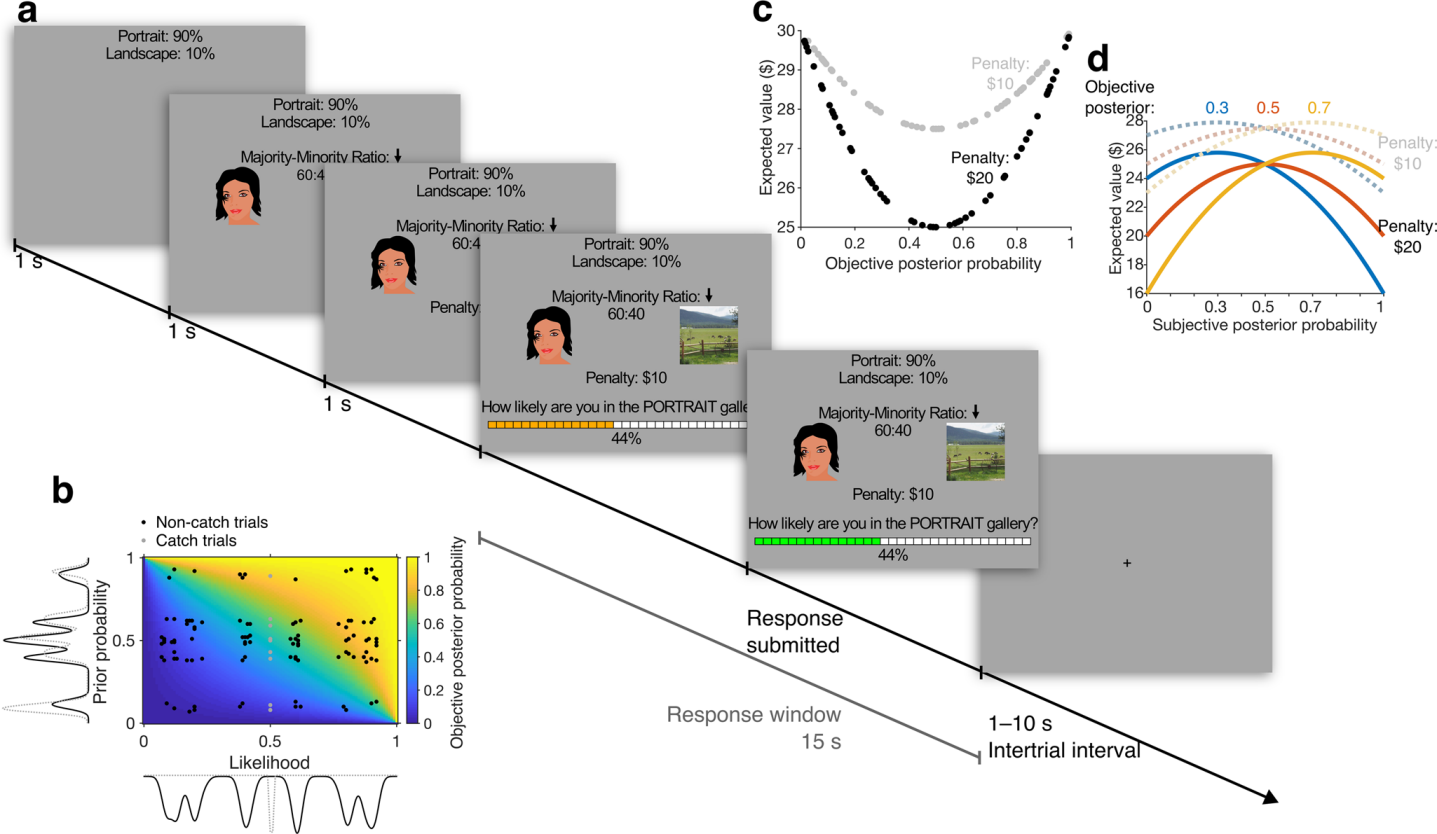
The trial structure and incentivization scheme of the Museum Inference Task. **a** Trial structure. Participants see the prior probability of being in a landscape or portrait gallery, one sample picture drawn from the gallery (indicated by the arrow above it); and the evidence strength, represented by the ratio of majority to minority pictures in the gallery. A decoy picture from the opposite category is shown to control for visual activations. Together, the sample and its strength determine the likelihood. The penalty reveals how much participants could lose from their endowment due to inaccuracy in their estimate. These trial elements could appear on screen in a variety of spatial or temporal orders. The face drawing (OpenCripart-Vectors via Pixaby, free to use under content license) is for visualization only. The actual face stimuli were photographs of human faces (see subsection “Image sets” in the “Methods” section). **b** The objective posterior probability of the questioned gallery conditional on the sample picture (colored grid) as a function of the prior probability of the questioned gallery (*y*-axis) and the likelihood of the sample conditional on the questioned gallery (*x*-axis), with points at prior–likelihood combinations that were used on trials during the scan session (non-catch trials: black circles, catch trials: gray circles). The catch trials’ likelihoods are plotted at 0.5 because catch trials omitted likelihood information, which, on this task, is equivalent to the likelihood equaling 0.5. **c** The expected value (EV) of a trial and the objective posterior probability of the questioned gallery are virtually uncorrelated on the task (trials with $10 penalty: gray circles, trials with $20 penalty: black circles). Thus, the incentivization scheme does not confound EV with posterior probability. **d** Curves depicting the relationship between trial EV and participants’ response (subjective posterior probability) when objective posterior probability is 0.3 (blue), 0.5 (orange), and 0.7 (yellow) and when penalty is $10 (dotted lines) and $20 (solid lines). The reward procedure incentivizes accuracy because EV increases as subjective posterior probability approaches the objective posterior probability. While EV decreases with penalty, submitting the objective posterior probability maximizes EV regardless of penalty.

The Museum Inference Task was a modified bookbag-and-poker-chip^5–7,9,13^ (or beads^15,24–27^) task that examined probabilistic inference from discrete samples of information. On each trial, participants estimated the posterior probability of being in one of two states—a portrait gallery that contained more pictures of faces than places or a landscape gallery that contained more pictures of places than faces. To make this estimate, participants were shown the prior probability of being in a state, a sample picture providing evidence with variable likelihood regarding the state, and the potential penalty for estimation inaccuracy (Fig. 1a). After viewing this information, participants were questioned about the posterior probability of being in one gallery (the questioned gallery) and reported their estimate—henceforth, “subjective posterior probability”—by moving a slider on a probability scale (Fig. 1a).

To determine how the prior and likelihood probabilities are integrated and individually contribute to the subjective posterior, we independently randomized each quantity. To this end, we created a set of 120 trials that tiled the joint probability space and ensured that the prior probabilities and likelihoods were uncorrelated (Fig. 1b; Pearson correlations, probabilities: *r* = 0.085, *p* = 0.359; logits: *r* = 0.104, *p* = 0.258). In individual trials, the prior probability was displayed as a percentage (e.g., 90% and 10% chance of being in the portrait and landscape galleries, respectively), while the likelihood was indicated by a majority–minority ratio^25,27^ (Fig. 1a). A more balanced majority–minority ratio (e.g., 60:40) indicated that the hidden gallery contained a relatively even mixture of images, and thus the sample picture provided weak evidence of the hidden gallery’s identity. In contrast, a more biased ratio (e.g., 90:10) indicated that the sample provided strong evidence about the hidden gallery. We interleaved 10 additional catch trials on which the sample picture and majority–minority ratio were omitted, and participants were to only report the prior probability. Catch trials were analyzed separately to verify that participants attended to the prior probability, but were not included in the fMRI analyses as they did not require Bayesian integration.

Moreover, to minimize value confounds, we used an incentive-compatible procedure inducing participants to provide accurate Bayesian estimates. First, to prevent serial trial effects, we did not provide participants with feedback on individual trials^7,9,28^; instead, participants were truthfully informed that trials were independent and their payment at the end of the session would depend on their accuracy on a randomly selected trial. Second, the payment for the selected trial was equal to a $30 endowment from which the trial’s inaccuracy penalty ($10 or $20) was deducted probabilistically, with the probability of a penalty being equal to the squared error between the true gallery and the participants’ posterior estimate. This scoring rule^23^ caused the expected value (EV) of a trial to be a U-shaped function of the objective posterior probability, removing linear correlations between the two variables (Fig. 1c; Pearson correlation coefficient: −0.007, *p* = 0.943). In addition, the scoring rule causing EV to increase as the subjective posterior approached the objective posterior (Fig. 1d, Supplementary Fig. 1), incentivizing participants to provide precise estimates. Note that this reward scheme is not intended to produce effects of the penalty on the participants’ probability estimates; instead, it incentivizes participants to veridically report the objective posterior at all levels of the posterior or penalty. The reward scheme and its implications were carefully explained to participants during the initial testing day (see the “Methods” section).

Participants first completed the task outside of the scanner (prescan session) and, if they met performance-based exclusion criteria (see the “Methods” section), were invited to return for a scan session in which they completed the task with a different set of trials under simultaneous fMRI. The scan session was divided evenly into four runs, with the questioned gallery alternating by run (Supplementary Data 1). At the start of each trial, the slider was reset to a random position to rule out confounds related to the motor report and to discourage participants from anchoring to any one reported probability. This decoupled the subjective posterior probability from the initial slider position and reduced its correlation with slider displacement to 0.55, well within the range that can be controlled for by linear models^29^.

### Probability estimates conform to approximate Bayesian inference

**Fig. 2:**
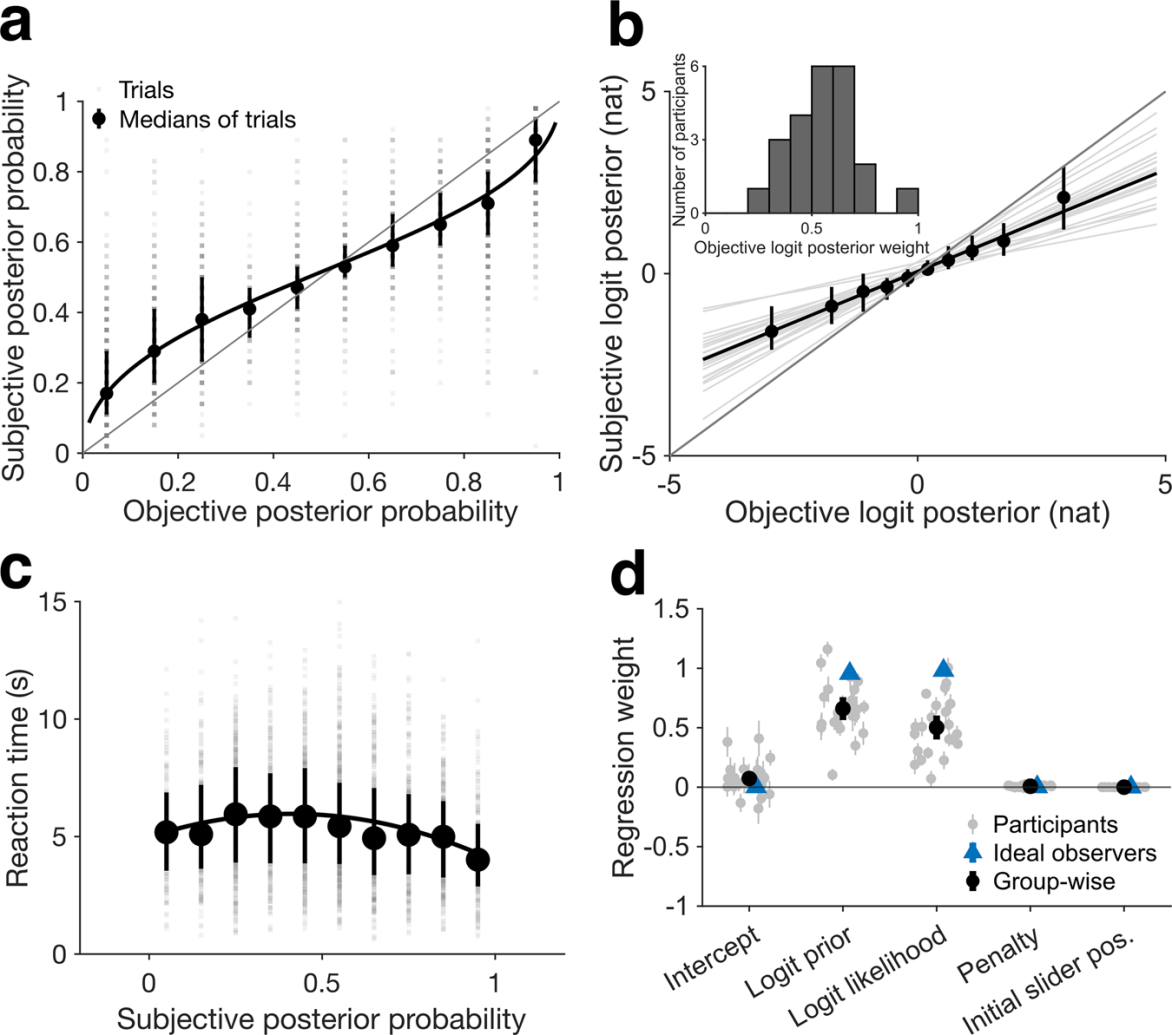
People closely estimate posterior probability by integrating described prior probabilities and likelihoods. **a** The subjective posterior probability (reported estimates) is monotonically associated with the objective posterior probability of the questioned gallery across all participants (*N* = 23) across all completed non-catch trials (gray squares). However, the subjective posterior is conservative (biased toward 0.5) compared to the objective posterior. For visualization, the median subjective posterior probability is binned by the objective posterior probability (black circles). The black curve is the regression line from **b** transformed into probability space. Error bars represent the interquartile range. The gray diagonal line represents unity. **b** **a** transformed into logit space. Subjective logit posterior is strongly associated with the objective logit posterior (*R*^2^ = 0.77, group-wise slope of objective logit posterior = 0.56, *p* < 0.001, linear mixed-effects model, Eq. (9)). Corresponding to the bias in subjective posterior probability in **a**, subjective logit posterior is conservative (biased toward 0) compared to the objective logit posterior. The black diagonal line is the least-squares regression line for the group while the thin, gray lines are the least-squares lines for each participant. For visualization, group median subjective logit posteriors are binned by objective logit posterior (black circles); however, the regression was carried out on the raw logits, not the binned medians. Error bars represent interquartile range. The long, thick, gray diagonal represents unity. In the inset is the distribution of objective logit posterior weights (slopes) across individual participants. **c** Reaction time peaks at intermediate subjective posterior probabilities and declines as subjective posterior approaches 0 or 1. Subjective posterior probability has a negative quadratic effect on reaction time after controlling for nuisance variables (i.e., the absolute value of slider displacement and the absolute difference between prior and likelihood) across all completed non-catch trials (gray squares). The black curve is the least-squares line for the individual trials. For visualization, median reaction time across all non-catch trials is binned by subjective posterior probability (black circles); however, the regression was carried out on the raw reaction times and probabilities, not the binned trials or trial medians. Error bars represent interquartile range. **d** Participants incorporate prior and likelihood into their subjective posteriors, but compared to simulated ideal observers (*N* = 23), they underweight both. Regression weights for individual participants (gray circles), at the group level over all participants (black circles), and at the group level over all simulated ideal observers (blue triangles). Error bars represent 95% confidence intervals. While all terms are statistically significant at the group level according to a linear mixed-effects model, penalty and initial slider position have negligible effects compared to logit prior and logit likelihood (Supplementary Table 1).

Twenty-three participants passed the exclusion criteria and were scanned using fMRI (see the “Methods” section). The subjective posterior probabilities that participants reported in the scanner increased monotonically with the objective posterior probability, suggesting that participants approximated Bayesian inference (Fig. 2a). A mixed-effects regression analysis (Eq. (5)) showed that the subjective posterior probability had a negative quadratic (i.e., inverse U-shaped) relationship with reaction time (after controlling for slider movement and the difference between prior and likelihood; fixed-effects coefficient of squared reaction time: −5.88, 95% confidence interval (CI): [−7.55, −4.21], *f*^2^ = 0.05, *N* = 23 participants, *T*(27.06) = −7.24, SE = 0.81, *p* < 0.001, Fig. 2c, degrees of freedom calculated with Satterthwaite approximation, see the subsection “Overview of behavioral modeling” of the “Methods” section). This suggests that reaction time increased as participants became more uncertain (i.e., subjective posterior approached 0.5), consistent with theory and experimental evidence that reaction time increases with decision difficulty^30,31^.

To examine our key hypothesis that participants integrated the prior and likelihood terms, we relied on the fact that Bayes’ theorem can be expressed as a sum of logits (log odds) of the prior and likelihoods—i.e., ${{{{{\rm{logit}}}}}}\left({{\rm {posterior}}}\right)={\beta }_{1}{{{{{\rm{logit}}}}}}\left({{{{{{\rm{prior}}}}}}}\right)+{\beta }_{2}{{{{{\rm{logit}}}}}}\left({{\rm {likelihood}}}\right)$, with ${\beta }_{1}={\beta }_{2}=1$ for perfect integration (Eqs. (7) and (8)). We thus modeled the subjective logit posterior as a weighted sum of the logit prior and logit likelihood (Eq. (10)) and analyzed the fitted coefficients ${\beta }_{1}{{{{{\rm{and}}}}}}\,{\beta }_{2}$ as measures of the weights that participants afforded to each term. Each term produced significant positive coefficients at the group level (Fig. 2d; fixed-effect logit prior weight: 0.66, 95% CI: [0.56, 0.76], *f*^2^ = 2.06, *N* = 23 participants, *T*(22.99) = 14.17, SE = 0.05, *p* < 0.001; logit likelihood weight: 0.50, 95% CI: [0.40, 0.60], *f*^2^ = 2.43, *N* = 23 participants, *T*(22.81) = 10.30, SE: 0.05, *p* < 0.001; Supplementary Table 1) and in each individual participant (Fig. 2d), indicating that the prior probability and likelihoods contributed independently to the subjective posterior. Further establishing the independent contributions of each term, the weights for logit prior and logit likelihood were uncorrelated across participants (Pearson correlation coefficient: −0.16, *p* = 0.46). Finally, models that contained only individual terms (i.e., logit prior, logit likelihood, or objective logit posterior) were inferior to those that contained terms for both logit prior and logit likelihood (Supplementary Fig. 2). The results were robust across the prescan and scan sessions, several methods of normalizing the data, and controlling for nuisance variables of penalty and initial slider position (Supplementary Table 1). Together, the findings confirm that participants did not rely solely on the prior or on the likelihood but integrated both terms to estimate the subjective posterior.

Although the subjective posterior estimates were consistent with Bayesian predictions, participants exhibited several systematic deviations from these predictions. First, participants showed a positive intercept, suggesting that they tended to overestimate the posterior probability of the questioned gallery (Fig. 2d; fixed-effect intercept: 0.07, 95% CI: [0.01, 0.13], *N* = 23 participants, *T*(23.82) = 2.59, SE = 0.03, *p* = 0.016, Supplementary Table 1). Additionally, the penalty coefficient was very small but significantly positive, indicating that participants noticed the penalty and reported slightly higher posterior probability estimates for the questioned gallery under a higher penalty (Fig. 2d, fixed-effect penalty coefficient: 0.01, 95% CI: [0.003, 0.013], *f*^2^ = 0.007, *N* = 23 participants, *T*(67.16) = 3.20, SE = 0.002, *p* = 0.002, Supplementary Table 1). However, consistent with an ideal observer’s response to our incentivization mechanism (Fig. 1d), the penalty did not significantly affect the accuracy of the subjective posterior (as calculated by the absolute deviation of the subjective from the objective posterior probability by participant; median difference between the deviations in the $20 versus $10 penalty conditions: −0.002, bootstrapped 95% CI: [−0.009, 0.015], *N* = 23 participants, sign statistic = 11, *p* = 1, paired sign test).

Finally, the most important departure from Bayesian integration was that, although the logit weights were significantly positive, they were significantly lower than those of the Bayesian ideal observers (Fig. 2d; difference in regression weights between individual participants and their corresponding simulated ideal observer, logit prior: −0.29, 95% CI: [−0.39, −0.20], Cohen’s *d* = −1.29, *N* = 23 participants, *T*(22) = −6.19, SD = 0.23, *p* < 0.001; logit likelihood: −0.48, 95% CI: [−0.58, −0.38]; Cohen’s *d* = −2.01, *N* = 23 participants, *T*(22) = −9.64, SD = 0.24, *p* < 0.001; paired *T*-test). The weights were strongly correlated between the prescan and scan sessions, suggesting that they were stable across our experiment (logit prior *r* across sessions = 0.77, *p* < 0.001; logit likelihood *r* across sessions = 0.76, *p* < 0.001; Pearson correlation).

Given these systematic departures from Bayesian integration, we considered whether participants may have used a non-Bayesian strategy to make their estimates—perhaps simply reporting the mean of the prior probability and the likelihood. To examine this possibility, we tested an additional group of participants on a control task (the Museum Averaging Task) that was identical in all respects to the Museum Inference Task but in which participants were asked to report the mean of two probabilities. The two sets of instructions elicited distinct strategies, as shown by quantitative model comparisons and model-free analyses of the numerical reports (Supplementary Fig. 3 and Supplementary Note 1); moreover, RTs peaked for intermediate probability estimates on the Inference but not on the Averaging Task (Supplementary Fig. 3). Together, these results provide converging evidence against the hypothesis that participants simply averaged the prior and likelihood probabilities strategy on the Museum Inference Task.

We further considered the possibility that, in the Museum Inference Task, participants performed a weighted average of the prior and likelihood (i.e., were better described by a weighted linear model of the two quantities) or a weighted average that considered the interaction between the two terms (i.e., were better described by a weighted linear model with interactions). We noted that the predictions of the latter model are very similar to those of Bayesian integration, as both models capture the integration of prior probability and likelihood (the linear model through the interaction term, and the Bayesian model by transforming the probabilities to log odds). Therefore, we partitioned the model space into two model families—the Bayesian and linear-interaction models on one hand, and the weighted average without interactions on the other—and calculated the family-wise exceedance probability, which relies on Bayes’ theorem to correct for the number of models within a family rather than simply adding the exceedance probabilities of the family’s constituent models^32^. The former model family had a much greater exceedance probability than the latter (0.83 vs. 0.17, respectively), showing that the participants’ strategy was better described by an interaction between prior and likelihood that is characteristic of approximate Bayesian inference.

Together, these findings argue against the hypotheses that participants used non-Bayesian strategies involving weighted or unweighted averaging of the probabilities. Thus, our finding that participants underweighted the logit prior and logit likelihood weights is consistent with previous findings showing that people approximate Bayesian integration by underweighting probabilities^28,33–35^, including priors^13^ and likelihoods^13^, particularly when the probabilities are conveyed through description as was the case in our task.

As a parsimonious measure of this underweighting, we thus computed the slope of the relationship between subjective and objective logit posterior (Eq. (9), Fig. 2b). This slope, which we refer to as the objective logit posterior weight, is equivalent to the probability weighting parameter from Prospect Theory^33^. The objective logit posterior weight was significantly > 0 (Fig. 2b, fixed-effect weight [group-level coefficient]: 0.56, 95% CI: [0.49, 0.63], *f*^2^ = 3.37, *N* = 23 participants, *T*(23.15) = 17.09, SE = 0.03, *p* < 0.001) but significantly lower than 1 (Fig. 2b, *N* = 23 participants, *T*(23.15) = −13.44, SE = 0.03, *p* < 0.001). Moreover, the slope was stable between the prescan and scan sessions (Pearson correlation coefficient: 0.81, *N* = 23 participants, *p* < 0.001), suggesting that it reliably captured inter-individual variability in the tendency to underweight the probabilities (Fig. 2b, gray lines and inset). Thus, we use the objective logit posterior weight as a measure of inter-individual variability in approximate Bayesian integration in our subsequent fMRI analyses.

### A region in left parieto-occipital cortex encodes subjective posterior probability

To search for candidate neural substrates of Bayesian integration, we modeled the fMRI signal during the decision period starting at the onset of the slider and ending at the participant’s response (Fig. 1a), and consistent with the behavioral analysis, we searched for BOLD responses to the probabilities in logit space. Note that the Bayesian prediction that the posterior probability is correlated with the prior and likelihoods precludes us from including all three terms into a single GLM model, as this would introduce severe multicollinearity. Given this strong constraint inherent to Bayesian logic, we adopted an alternative strategy of first searching for regions where the BOLD signals scaled positively with the subjective logit posterior, and then analyzing if these regions *separately* encoded both the logit prior and logit likelihood consistent with Bayesian integration.

**Fig. 3:**
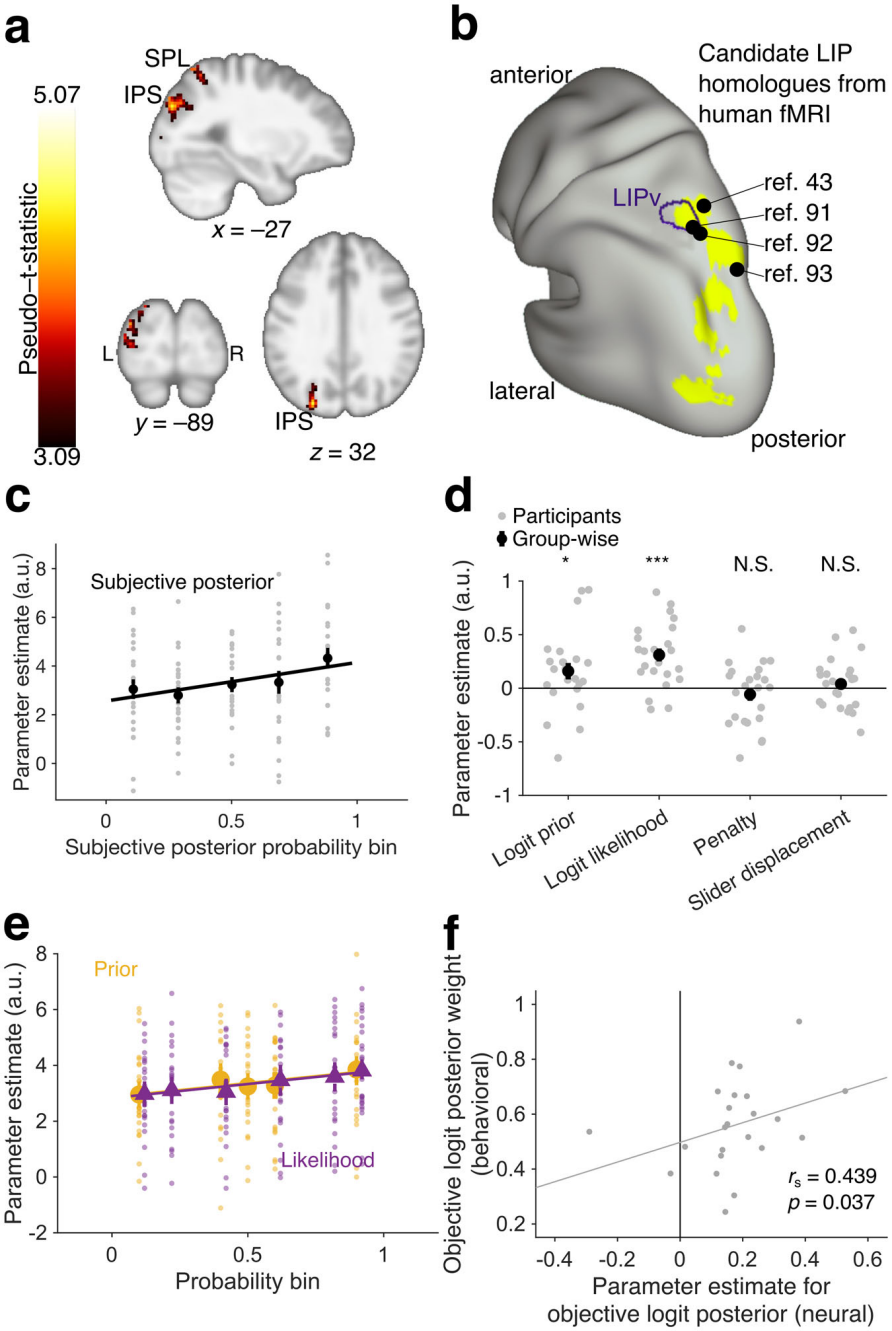
A left-hemisphere cluster mostly within posterior parietal cortex (PPC) and extending into anterolateral occipital cortex encodes subjective posterior beliefs about the questioned gallery. **a** Activation in one cluster spanning superior parietal lobule (SPL) and intraparietal sulcus (IPS) in left PPC and left anterolateral occipital cortex tracks the subjective logit posterior of the questioned gallery, making it a candidate posterior belief–encoding region. Cluster-level familywise error (FWER)-corrected *p* = 0.003 (permutation test based on cluster-defining height threshold of *p* = 0.001, *N* = 23 participants). The anatomical template was smoothed at a full width at half maximum (FWHM) of 5 × 5 × 5 mm for visualization purposes^89^. **b** Surface rendering of the cluster in **a** shows that it overlaps candidate human homologs to the lateral intraparietal (LIP) area, a region in monkey PPC that has been implicated in Bayesian integration of probabilistic information^46,47,49,52^. The purple outline corresponds to the human homolog to ventral LIP^90^. Points represent candidate human homologs of LIP according to task-based fMRI studies in humans^43,91–93^. Rendered in Connectome Workbench^94^. **c** Corroborating results in **a**, mean activation of the cluster visually increases with binned subjective posterior probability (binned in fifths). The black circles represent the mean of participants’ parameter estimates for subjective posterior probability within the cluster; error bars represent standard error. The gray circles represent the individual participants’ parameter estimates. The least-squares line of individual participants’ binned parameter estimates is shown to visualize the increase in activation with subjective posterior, but neither this plot nor this analysis was used to test for any effect. Despite the impression of a slight nonlinearity, the effect of subjective posterior was still significantly positive after excluding trials in the highest bin (*p* = 0.024), and a quadratic effect of subjective logit posterior was not statistically significant (*p* = 0.064). **d** Post-hoc analyses of the cluster in **a** show it is significantly positively associated with both the logit prior of the questioned gallery and the logit likelihood of the sample picture conditional on the questioned gallery, after accounting for the inaccuracy penalty and slider displacement on the trial (gray circles: individual participants, black circles: group-level results). Error bars represent standard error. *p < 0.05, ***p < 0.001. Even though the distribution of parameter estimates for the logit prior was not significantly different from normal (*N* = 23 participants, *p* = 0.098, Lilliefors test), a nonparametric analysis also supported the positive group-level effects of the logit prior (signed rank = 208, *N* = 23 participants, *p* = 0.033). **e** Corroborating the results in **d**, activation of the cluster visually increases with binned prior probability (yellow circles) and likelihood (purple triangles). The darker points represent the mean of participants’ parameter estimates for prior probability and likelihood, respectively; error bars represent standard error. The translucent points represent the individual participants’ parameter estimates. The least-squares lines of individual participants’ binned parameter estimates are shown to visualize the increase in activation with prior (yellow line) and likelihood (purple line), but they were not used to test for an effect. Prior and likelihood were binned into the same groups by which they had been binned when the session parameters were set (marginal histograms in Fig. 1b, see also the “Methods” section). Three individual points are not shown for clarity of visualization. The data points for likelihood have been shifted to the right by 0.02 to reduce the visual overlap with the data points for prior probability. **f** Parameter estimate for BOLD signal tracking objective logit posterior within the cluster is positively correlated with behavioral objective logit posterior weight across all participants (Spearman correlation: 0.439, *N* = 23 participants, *p* = 0.037), suggesting that distortions in neural representations of posterior probability in the cluster contribute to the degree of distortion in participants’ subjective posterior probabilities. Each point represents one participant. The gray line is the least-squares line.

To identify regions encoding the subjective logit posterior, we used a whole-brain general linear model (WB-GLM 1) that contained subjective logit posterior as a predictor and controlled for subjective posterior certainty (the absolute value of the subjective logit posterior), motor preparation for hand or eye movements (initial slider position), and reward expectation (penalty and EV based on subjective posterior probability; Eq. (4)). This analysis revealed one cluster tracking subjective logit posterior that spanned parts of the superior parietal lobule (SPL) and intraparietal sulcus (IPS) in the left posterior parietal cortex and extended partially into left anterolateral occipital cortex (Fig. 3a, b, Supplementary Tables 2 and 3, cluster-level familywise error–corrected *p* = 0.003, permutation test). The signal in this cluster increased monotonically as a function of subjective posterior probability (Fig. 3c; data binned for visualization). Additional analyses ruled out non-monotonic patterns, by showing that the effect of subjective logit posterior was still significantly positive after excluding trials in the highest bin (see subsection “fROI analyses for the Museum Inference Task” in the “Methods” section; parameter estimate: 0.16, 95% CI: [0.02, 0.29], *f*^2^ = 0.05, *N* = 23 participants, *T*(66.01) = 2.30, SE = 0.07, *p* = 0.024), and a quadratic effect of subjective logit posterior was not statistically significant (parameter estimate: 0.15, bootstrapped 95% CI: [0.09, 0.21], signed rank = 199, *N* = 23 participants, *Z* = 1.86, *p* = 0.064; Wilcoxon signed rank test used since parameter estimates for subjective logit posterior were not normally distributed). We found subthreshold activation but no significant clusters when we tested the objective (WB-GLM 2) instead of the subjective posterior (Supplementary Fig. 4).

To verify if the activity in the parieto-occipital cluster explained individual differences in approximate Bayesian integration, we used the weight (slope) of the subjective versus the objective logit posterior as noted above (Fig. 2b; Eq. (9)). To obtain an analogous measure of neural probability weighting, we re-analyzed the activity in the ROI using a GLM with a term for the objective logit posterior while controlling for the nuisance regressors of penalty and slider displacement (fROI-GLM 1). The behavioral weights and neural parameter estimates for objective logit posterior were positively correlated across participants (Spearman *ρ* = 0.439, bootstrapped 95% CI: [0.070, 0.714], *p* = 0.037; Fig. 3f). Thus, deviations in the cluster’s activation with respect to the objective posterior probability predicted the degree of systematic distortion (conservatism) of participants’ reported estimates (Fig. 2a).

In the second analysis step, we examined if the cluster encoding the subjective posterior had independent responses to the prior and likelihood. This analysis is crucial for excluding the null hypothesis that the encoding of subjective posterior reflects tracking of only one quantity (either the prior or likelihood), rather than independently tracking both as required for Bayesian integration. Consistent with the latter hypothesis, analysis of the cluster’s activity with a GLM that had separate terms for logit prior and logit likelihood (fROI-GLM 2) produced significant and positive parameter estimates for each term (logit prior parameter estimate: 0.159, 95% CI: [0.035, 0.283], *f*^2^ = 0.07, *N* = 23 participants, *N* = 23 participants, *T*(92) = 2.55, SE = 0.06, *p* = 0.013; logit likelihood: 0.310, 95% CI: [0.187, 0.434], *f*^2^ = 0.27, *N* = 23 participants, *N* = 23 participants, *T*(92) = 4.98, SE = 0.06, *p* < 0.001; Fig. 3d, e; Supplementary Table 4). We further asked whether this analysis may have been biased to detect significant effects of both terms given the cluster’s scaling with subjective posterior probability. To rule out this hypothesis, we conducted a permutation analysis in which we randomized the labels of the logit prior and logit-likelihood terms (Supplementary Note 2, Supplementary Fig. 5). This procedure holds the logit posterior constant, and provides a null distribution of the GLM parameters that are expected only from an encoding of the logit posterior without a true encoding of the logit prior and logit likelihood terms. The true (non-randomized) GLM parameters for the prior and likelihood were entirely outside their respective null distributions, ruling out the hypothesis that they were merely epiphenomena of a representation of the posterior (Supplementary Fig. 5).

Additional analyses confirmed this conclusion. Because the subjective posterior was equivalent with the final slider position, the above analyses rule out a mere encoding of the final slider position. Moreover, slider displacement and penalty were included as nuisance parameters in the GLM and produced nonsignificant parameter estimates (slider displacement: 0.04, 95% CI: [−0.08, 0.16], *f*^2^ = 0.004, *N* = 23 participants, *T*(92) = 0.63, SE = 0.06, *p* = 0.53; penalty: −0.06, 95% CI: [−0.18, 0.07], *f*^2^ = 0.009, *N* = 23 participants, *T*(92) = −0.921, SE = 0.06, *p* = 0.360), ruling out reward or sensorimotor confounds. A separate conjunction analysis showed that the parieto-occipital cluster overlapped with a significant cluster showing a conjunction of logit prior and logit-likelihood effects (Supplementary Fig. 6; Supplementary Table 5). Finally, the cluster showed positive effects of both prior and likelihood (*T*-stats > 0 for both terms) in a majority of individual participants (15 of 23), ruling out the possibility that the two quantities were encoded in different participant groups. Together, these findings suggest that the parieto-occipital cluster provided independent encoding of both the prior and the likelihood, consistent with Bayesian integration.

### Lack of consistent category-specific representations of probability

**Fig. 4:**
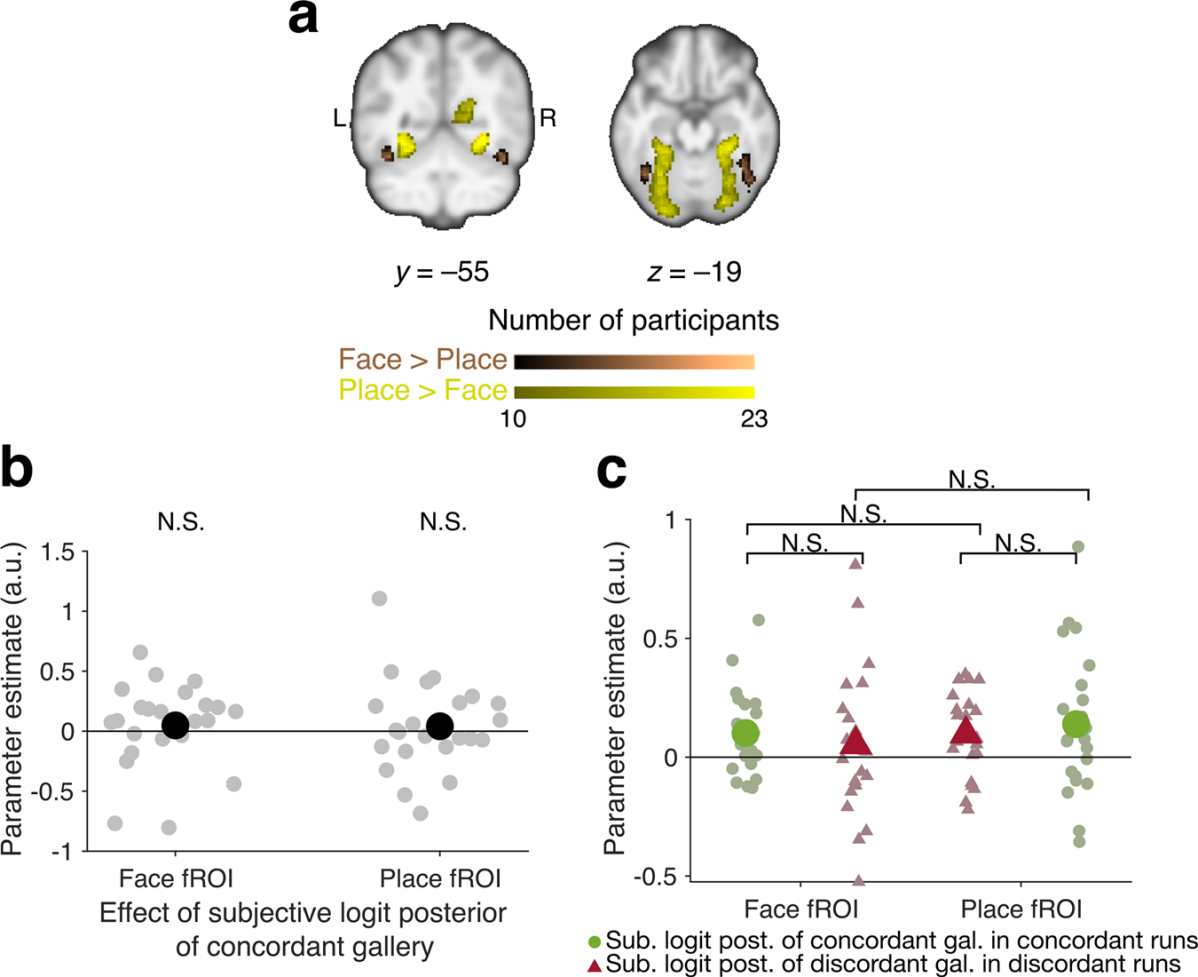
Lack of evidence that face- and place-selective functional regions of interest (fROIs) encode subjective posterior beliefs about category-concordant galleries (i.e., the galleries corresponding to the preferred stimuli of that region: portrait gallery corresponding to face fROI and landscape gallery corresponding to place fROI). **a** Participant-wise (*N* = 23 participants) overlap of face- (brown) and place- (yellow) selective fROIs from an independent functional localizer task, normalized to MNI space for visualization. Within participants, voxels were thresholded in native space at an uncorrected *p*-value of 0.001 for the respective contrasts (Face > Place or Place > Face). Face-selective fROIs encompass the fusiform face area and place-selective fROIs encompass the parahippocampal place area. The anatomical template was smoothed at a FWHM of 5 × 5 × 5 mm for visualization purposes^89^. **b** Neither the face-selective nor the place-selective regions show significant effects of the subjective logit posterior of their concordant galleries. Because there were only two galleries (portrait and landscape), their posterior probabilities were complementary. Group-level statistics are in black; participant-level statistics are in gray. **c** After dividing trials by their questioned galleries (portrait or landscape), neither fROI showed preferential activation tracking the posterior probability of its concordant gallery and neither posterior probability had a higher parameter estimate in its concordant fROI. Group-level statistics are in saturated colors while participant-level statistics are in pastel colors. Statistics for concordant galleries are green circles while statistics for discordant galleries are red triangles. Between-participant error bars are the standard error of the mean of the participants’ parameter estimates. On most points, the error bars are too small to be visible. NS not significant.

Previous studies suggest that, when probabilistic outcomes are yoked to category-specific visual inputs (e.g., face or place images as probabilistic evidence), probabilistic reasoning engages category-specific areas^36^ or immediately adjacent regions^19^. To determine if this were the case on our task, we used an independent face–place localizer (see the subsection “Face–Place Localizer” in the “Methods” section) to identify participant-specific face- and place-selective fROIs (Fig. 4a). Neither fROI showed significant encoding of the category-concordant subjective logit posterior when all runs were considered together (Fig. 4b; Supplementary Fig. 7a; Supplementary Table 6, fROI-GLM 3 in the “Methods” section), or when they were separated according to the concordance between the questioned gallery and the fROI’s preferred category (Fig. 4c; test statistic for interaction between cluster and fMRI contrast in ANOVA: *F*(3) = 0.84, *η*^2^ = 0.01, *p* = 0.47; see also Supplementary Fig. 7d; Supplementary Table 8). Analyses of prior and likelihood activations (fROI-GLM 2) found a significant response only to the logit likelihood of the portrait gallery in the face fROI (parameter estimate: 0.170, 95% CI: [0.050, 0.290], *f*^2^ = 0.028, *N* = 23 participants, *T*(276) = 2.78, SE = 0.06, *p* = 0.006), but no other significant responses in the face or place fROIs (Supplementary Fig. 7b, c, e, f, Supplementary Tables 7 and 9). Likewise, category-specific whole-brain analyses showed no consistent results, with no significant clusters tracking probabilities with respect to the portrait or landscape gallery except one tracking the subjective logit posterior (Supplementary Fig. 8, Supplementary Table 10) and another tracking the logit prior (Supplementary Fig. 9; Supplementary Table 11). Together, these findings suggest that responses to category-specific probabilistic information were not pronounced in our task.

## Discussion

To elucidate neural substrates for Bayesian integration, we designed a task in which participants were incentivized to report accurate estimates of the posterior probability of one of two hidden states, based on the integration of the prior probability and likelihood of the evidence regarding the state. fMRI analyses revealed a cluster that straddled the left posterior parietal and anterolateral occipital cortex and tracked the subjective posterior probability, and crucially, both of its components of prior probability and likelihood, independently of sensorimotor confounds or expected value. The sensitivity to subjective posterior probability in the parietal–occipital cluster correlated with individual behavioral sensitivity, suggesting that the cluster modulates inter-individual variability in probability weighting. These results add to our understanding of the neural mechanisms of probabilistic inference and highlight a region of the human parieto-occipital cortex as a candidate substrate for the integration of prior and likelihood into a subjective representation of posterior probability.

Our experimental design was inspired by axiomatic approaches to identify the representations of distinct quantities that comprise reward prediction errors^37,38^ and is distinguished from the previous literature by its focus on the neural substrates of prior and likelihood integration. While many studies of decision-making involve probabilistic inference, these studies have typically focused on economic or information sampling decisions that rely on the results of the inference process rather than on the mechanisms of inferential computations per se (e.g., the Beads Task^15,25,26^). Several neuroimaging studies did focus on probabilistic inference and reported activations in subcortical^17^, frontal^17,19–21^, temporal^19,22^, and parietal regions^20–22^; however, these studies parametrically manipulated either the prior probability or the likelihood while holding the other quantity constant, precluding them from identifying areas involved in Bayesian integration. An elegant study by Vilares et al. ^16^ factorially modulated prior and likelihood *uncertainty* but focused more on the distinct encoding of these quantities rather than their integration into posterior probability. In contrast with our approach of conveying prior and likelihood using a common numerical format to facilitate analysis of their neural integration, Vilares et al.’s design required different strategies to glean the prior and the likelihood uncertainties (experience-based learning and visual, respectively), which may have contributed to their findings of mainly distinct neural representations of these two quantities.

Ting et al. ^11^ also parametrically modulated prior and likelihood; however, they required participants to decide which of two options was more likely to be rewarded, equating neural representations of posterior probability with EV. In contrast, in our study, participants reported the probability of one of two states that were not defined by the prospect of reward, and they were incentivized to maximize accuracy at all levels of posterior probability, ensuring that neural representations of posterior probability did not reflect EV. Our goal to localize neural representations of posterior probability irrespective of EV thus differed from that of previous authors who studied how reward and probability are combined to make decisions^15,39^. Our results are consistent with imaging studies in humans^20^ and neural recordings in monkeys^40–42^ showing that areas of the parietal cortex can encode probabilistic information irrespective of reward. In particular, the section of the parieto-occipital cluster in IPS overlaps with the human homolog to monkey lateral intraparietal (LIP) area^43–45^, which has been further associated with probabilistic inference in support of decision-making^46,47^ and was recently shown to contain separate representations of likelihood^40^ and prior uncertainty^41^. Together with this literature, our results thus point to parieto-occipital cortex as an important neural substrate for inferential reasoning and probabilistic sophistication, the ability to reason about probability independently of value^20,48^.

In previous studies in humans and monkeys, sensory evidence for categorical decisions indicated by a property of a sensory stimulus—e.g., the coherence of a random-dot motion stimulus^46,49^—was encoded in sensory areas selective for the relevant stimulus, such as motion-selective areas. More relevant to our study, Philiastides et al. ^19^ associated different likelihoods with specific images of faces or houses and found that these likelihoods were encoded in the ventral temporal lobe near face-selective and place-selective visual regions, respectively. In our experiment, in contrast, the likelihood was described numerically while images of faces and places merely cued a stimulus category. Rather than activating areas selective to faces or places, this more abstract presentation modality—which has been intensely studied behaviorally^5–7,9,13,33,34,50,51^ but less so with neuroimaging^18^—elicited consistent representations of the probability of the questioned gallery only in the parieto-occipital cluster. Together with evidence that monkey parietal neurons are sensitive to probabilistic information even when probabilistic information is conveyed through learned cues^40,41,47,52^, these findings suggest a domain-general involvement of parietal-occipital areas in probabilistic inference, regardless of whether concrete sensory evidence is conveyed from sensory regions (during perceptual decisions) or whether more abstract—e.g., numeric—information is directly encoded in higher-order parieto-occipital regions (as in our current task).

Notably, participants systematically overestimated the posterior probability of the questioned gallery when it was close to 0 and underestimated it when it was close to 1. This behavior, described by a phenomenon called probability weighting, or approximate Bayesian inference (Eq. (10))^13^, is typically observed in humans’ judgments from described probabilities^13,28,33–35^. Recent studies suggested that inter-individual variations in probability weighting represent optimal adaptations to variable levels of cognitive imprecision in the representation of probabilistic quantities^53–56^, although this hypothesis requires validation in future studies with larger participant samples. Whatever the explanation, however, we show that participants’ posterior probability weighting was positively correlated with their neural parameter estimates for posterior probability within the parieto-occipital cluster, suggesting that the parieto-occipital cortex at least partially mediates the mechanisms of posterior probability weighting.

These findings, in turn, raise questions about the relationship of our results and human parietal activations when people solve arithmetic problems through (explicit) mental calculation^57,58^. The findings from the control Museum Averaging Task strongly suggest that participants did not merely adopt an averaging heuristic on the Inference Task. Moreover, while arithmetic-related parietal activation increases with problem complexity or difficulty^58–61^, in the Inference Task, decision difficulty (as indexed by reaction times) covaried with subjective posterior certainty, which was orthogonal to the subjective posterior probability tracked by parieto-occipital activations.

While these results rule out a simple explanation based on averaging, an important question for future research is how the parietal-occipital cortex may integrate numeric quantities of prior and likelihood. Based on single-neuron recordings in the monkey LIP^40,41^ and findings of distinct encodings of probabilistic quantities in this area^40,41^, we hypothesize that prior probability and likelihood can be encoded by distinct populations of cells and integrated into a representation of posterior probability by the local parietal circuitry when all relevant information is conveyed in an abstract format. A recent study by Luyckx et al. ^62^ found that representational patterns for numbers are used in a bandit-learning task, suggesting that abstract stimuli conveying probabilistic information may be mapped onto a magnitude scale that is normally used to represent numbers. Yet this mechanism involved a multidimensional representation in principle inconsistent with our univariate results. Nevertheless, the broader hypothesis that the brain approximates Bayesian inference using a relational magnitude representation remains worthy of future investigation.

## Methods

### Participants

For the Museum Inference Task, 44 healthy, right-handed participants (17 females) were recruited through fliers posted on the Columbia University campus and through the recruitment system for the Columbia Business School Behavioral Research Lab. This pool consisted of Columbia University students, other Columbia affiliates, and affiliates of other universities in the New York Metropolitan Area, and they did not report any psychiatric or neurological disorders. Participants first completed a session outside of the scanner (prescan session); 14 participants were not allowed to advance to the scan session because their responses during the prescan session reflected disengagement or lack of comprehension (see the subsection “Performance-based exclusion criteria”). Another participant was excluded because of excessive motion inside the MRI scanner, and six participants withdrew from the study. As a result, the final sample consisted of 23 participants (8 females).

For the Museum Averaging Task, 22 healthy, right-handed participants (13 females) were recruited through social media and email, including current students at Columbia University Medical Center. Participants did not report any psychiatric or neurological disorders. Participants completed one session of the Museum Averaging Task at a computer just as the other cohort had completed the prescan session of the Museum Inference Task; there was no scan session. Three participants’ data were excluded from the analysis because their responses reflected disengagement or lack of comprehension as indicated by our performance-based exclusion criteria, leaving 19 remaining participants (11 females). Performance-based exclusion criteria for the Averaging Task were the same as those for the Inference Task except they were adapted to the Averaging Task: the comprehension quiz was tailored to the Averaging Task, minimal sensitivity to the Gallery 1 Probability was tested instead of minimal sensitivity to prior probability, minimal sensitivity to the mean of the Gallery 1 and Gallery 2 probabilities was tested instead of minimal sensitivity to objective posterior probability, and no information-sampling task was administered (see the subsection “Performance-based exclusion criteria”).

All relevant ethical regulations were followed, and all participants provided signed informed consent. Experimental procedures for the Inference and Averaging tasks were approved by the Institutional Review Boards at Columbia University and the New York State Psychiatric Institute, respectively.

### Statistics and reproducibility

The final sample consisted of 23 participants. Data were analyzed in MATLAB (versions R2018b, R2021a, and R2022a). In behavioral analyses, wherever possible, we implemented linear mixed-effects regression to properly account for between-participant (fixed-effects) variance and within-participant (random-effects) variance, using the MATLAB function fitlme with maximum-likelihood estimation. To acquire first-level (participant-level) fMRI data, we used the general linear model (GLM) framework implemented in SPM12, Version 7487 (https://www.fil.ion.ucl.ac.uk/spm). To produce second-level (group-level) whole-brain maps, we used SnPM13.1.08 (http://nisox.org/Software/SnPM13/)^63^ applying a cluster-wise correction for multiple comparisons using non-parametric permutation tests. All reported *p*-values are two-sided except the cluster-level, familywise error–corrected *p*-value of the parieto-occipital cluster (Fig. 3a, b). Further detail is provided below, especially in the subsections “Overview of behavioral modeling” and “fMRI data analysis overview”.

### Experimental sessions

The full study took place over a prescan and a scan session scheduled on different days. Both sessions included the Museum Inference Task (the primary behavioral task in this study; Fig. 1a) while the scan session additionally included a Face–Place Localizer Task. We wrote all tasks in MATLAB using the Psychtoolbox extensions^64,65^.

The prescan session was administered on a computer outside of the scanner. Participants first viewed a narrated slideshow on the instructions for the Museum Inference Task. They were also administered comprehension quizzes on the instructions, which they had to pass before proceeding (see the subsection “Performance-based exclusion criteria”). After passing the instructions quiz, participants completed 10 practice trials of the Museum Inference Task to familiarize themselves with the relationship between response accuracy and the probability of being penalized while avoiding overtraining. Each practice trial was followed by a corresponding mock payout trial to show participants what they could have earned from that trial in the main task based on their submitted estimate if the trial had been chosen for payout; however, these practice trials did not affect the participants’ earnings. Then, participants completed the Museum Inference Task, after which their performance was evaluated to determine if they met the remaining performance criteria to advance to the scan session; if not, they were removed from the study.

In the scan session, participants watched a summarized version of the instructions slideshows before completing the Museum Inference Task and the Face–Place Localizer in the MRI scanner. Participants were debriefed at the end of the session.

### Estimation Stage of the Museum Inference Task

The Museum Inference Task consisted of an Estimation Stage followed by a Payout Stage. To encourage participants to remain engaged with the task, we designed the task so that participants’ estimation accuracy influenced their earnings. During the Estimation Stage, participants estimated the posterior probability of a hidden state depicted as a museum gallery. During the Payout Stage, one trial was drawn at random to determine the participant’s payout. At the beginning of each session, the participant was given a $30 endowment from which a penalty of $10 or $20 would be withdrawn during the Payout Stage depending on the deviation of the participant’s estimate from the eventual outcome. We based participants’ earnings on a single estimation trial instead of averaging potential earnings across all estimation trials to discourage participants from allowing their accuracy to decline during later trials if they had believed their performance on earlier trials had been sufficient to make high earnings.

The Estimation Stage of the Museum Inference Task consisted of 130 trials divided into 4 runs of 32, 33, 32, and 33 trials, respectively. On each trial, participants had to estimate the posterior probability of being in either a portrait gallery that contained more pictures of faces than places or a landscape gallery that contained more pictures of places than faces. Participants viewed the prior probability of being in each gallery and possibly also the likelihood of the sample picture. On 10 catch trials distributed randomly through the Estimation Stage, the sample picture and likelihood were absent, so participants would have to estimate the posterior probability with the prior probability only (Fig. 1b). We inserted these catch trials to ensure that participants paid attention to the prior probability (see the subsection “Performance-based exclusion criteria”), and they were not included in the behavioral or fMRI analyses.

The prior probability was displayed as a percentage (e.g., 90%). The likelihood information consisted of one face picture^66^, one place picture^67^, and potentially the majority-to-minority ratio of pictures in the hidden gallery (e.g., 60:40). One face picture and one place picture were always shown on each trial to control for the fMRI activation by the appearance of faces and places, as we were instead interested in the degree of potential face- and place-selective activation by probabilistic information. During non-catch trials, the likelihood would consist of a majority–minority ratio of picture types in the hidden gallery, one sample picture randomly drawn from the hidden gallery, and one decoy picture which signaled the opposite category from the sample picture (i.e., if the sample picture were a face, the decoy would be a place and vice versa) (Fig. 1a). An arrow appeared over the true sample picture so that participants could distinguish it from the decoy picture (Fig. 1a). During catch trials, in place of the likelihood, there were two decoy pictures and no majority–minority ratio. Participants were also shown the penalty that they could lose from the endowment if the trial was chosen for payout (see “Payout trial”).

A trial began with the prior probability, likelihood information, or penalty appearing (trial components) over a gray background (Fig. 1a). The prior probability, likelihood (or likelihood decoy), and penalty appeared one at a time with the first component appearing at the instant of trial start and each succeeding component following the previous component by 1 s (Fig. 1a). The trial components’ spatial order of appearance was stable throughout the prescan and scan sessions but counterbalanced by participant so that participants could expect the information to be in the same place while allowing us to control for potential effects of spatial order. The trial components’ temporal order of appearance was randomized by trial to control for potential primacy and recency effects. Effects of temporal order on reported probability estimates (subjective posterior) were negligible and are not discussed further.

Participants completed a trial by reporting their estimate of the posterior probability of the questioned gallery (the gallery in the prompt below the slider) by using a trackball to move a slider that appeared at the bottom of the screen 1 s after the last trial component. The initial slider position was randomized on each trial to reduce the correlation between slider movement and reported posterior—facilitating the separation of the potentially confounding effect of slider movement from the task variables of interest—and to discourage participants from anchoring to any one reported probability. (Randomizing the initial slider position reduces the correlation between slider displacement and reported posterior from nearly 1 to 0.55 across all completed trials in the scan session.) The slider remained on screen for 15 s (“response window,” Fig. 1a). We chose a response window of 15 s because it was the shortest response window that captured approximately 80% of responses from 80% of participants during piloting. The selected posterior probability estimate was indicated by the amount of the slider from left to right that was highlighted in orange and by an explicit percentage below the slider. Both these indicators were updated in real-time. To account for potential framing effects induced by the prompt, the questioned gallery was the portrait gallery on the first and third runs while it was the landscape gallery on the second and fourth runs. The slider was divided into 33 discrete posterior probability bins, increasing in steps of 3% from 2% on the left to 98% on the right. We chose these increments to discourage participants from anchoring to “round” numbers (e.g., multiples of 10% or 25%) and so that submitted posterior estimates could not be 0% or 100%, which would make the behavioral model inestimable (see the subsection “Modeling subjective posterior probability”). The participant confirmed their response by clicking a button on the trackball, after which the highlighted section of the slider would change colors from orange to green to indicate that the response had been recorded. The screen remained unchanged until the end of the response window plus 0.5 s. If the participant did not submit a posterior probability estimate within the 15-s response window, instead, the slider would freeze for 0.5 s and the percentage below the slider would be replaced by text reading, “Estimate not submitted.” To encourage participants to respond within the response window, participants were truthfully warned that if a response was missing from a trial that happened to be chosen for payout, they would automatically lose that trial’s penalty. Across all participants during the scan session, only 10 trials had omitted responses (all of which were non-catch trials), with 3 participants missing one trial, 2 participants missing two trials, and 1 participant missing three trials.

Each estimate trial was followed by an intertrial interval during which a small, black fixation cross appeared over the gray background (Fig. 1a). To maximize the efficiency of parameter estimation for the general linear models in the fMRI analysis, the duration of each intertrial interval was drawn from an exponential distribution with mean 3.5 s, truncated with a lower bound of 1 s and an upper bound of 10 s^68^.

Since the task was designed to investigate prior–likelihood integration after receiving only one sample, we sought to prevent behavioral artifacts from serial trial effects such as the gambler’s fallacy. Therefore, we truthfully told participants that each estimation trial was independent of all other estimation trials, and the identity of a trial’s hidden gallery was never revealed during the Estimation Stage.

To determine the set of prior probabilities and majority–minority ratios used for the non-catch trials in each session, we randomly sampled 60 trials from discrete bins that we established for prior probability (0.1, 0.4, 0.5, 0.6, and 0.9, arbitrarily chosen as the prior of the portrait gallery) and majority–minority ratio (60:40, 80:20, and 90:10). Majority–minority ratios represented evidence strength $$\theta$$, which was defined on the interval $$0.5 < \theta \le 1$$ and corresponded to the numerator of the majority–minority ratio divided by 100. A random jitter (−0.03, −0.02, −0.01, 0, 0.01, 0.02, or 0.03) was then added to each prior probability and evidence strength with equal probability. A “true” hidden gallery was assigned to each trial based on the prior probability of the portrait gallery (e.g., if the prior probability was 0.6, there was a 60% chance the trial’s hidden gallery would be a portrait gallery and a 40% chance it would be a landscape gallery). A trial’s sample picture was assigned to signal the hidden gallery with a probability equal to the trial’s evidence strength (e.g., there was a 60% chance that the sample would be a face on a trial on which the hidden gallery was the portrait gallery and the evidence strength was 0.6). These 60 trials were duplicated for each condition of inaccuracy penalty ($10 or $20). The parameters for the remaining 10 catch trials were assigned by assigning two trials to each of the five prior probability bins (one trial for each penalty condition) and jittering the prior probabilities by the aforementioned jittering method. The order of the trials was then randomly permuted, and the session was separated into four runs, with 32 trials in the first and third runs and 33 trials in the second and fourth runs. Supplementary Data 1 contains a list of parameters for each estimation trial in the scan session. Figure 1b displays the prior–likelihood combinations for the scan session, with the result of the binning and jittering process visible as peaks on the kernel density plots against each axis.

### Payout Stage of the Museum Task

After the Estimation Stage was complete, one estimation trial was chosen at random with equal probability to determine the participant’s payment. This trial was displayed along with its reported posterior probability estimate from the Estimation Stage. If the participant had failed to report a posterior probability estimate on that trial, the participant was notified that the inaccuracy penalty would be automatically subtracted from their endowment, and the session would end. Otherwise, the trial’s hidden gallery was revealed, and the participant was told whether they would keep all their endowment or if they had lost the error penalty, depending on the posterior probability estimate that they had submitted during the Estimation Stage.

### Binarized scoring rule

We used a binarized scoring rule with a quadratic loss function^23^ to determine *p*_loss_, the probability that the participant would lose the penalty on each trial. Specifically, *p*_loss_ was given by Eq. (1),

1 $${p}_{{{\rm {loss}}}}={\left(I-\pi \left(Q|x\right)\right)}^{2}$$

where $\pi ({Q|x})$ is the participant’s report of the posterior probability and *I* is an indicator of whether the “true” hidden gallery was the questioned gallery on that trial (1 if yes, 0 if no). Therefore, the quantity ${\left(I-\pi \left(Q|x\right)\right)}^{2}$ is a measure of the participant’s error—the difference between the hidden gallery and the participant’s estimate of the probability of being in that gallery. Thus, ${p}_{{{\rm {loss}}}}$ is minimized when $\pi ({Q|x})$ is the objective posterior probability of the questioned gallery according to Bayes’ theorem because the probability that the hidden gallery is the questioned gallery is expressed by this objective posterior probability $\Pr \left(Q|x\right)$ (Eq. (6)). Manipulating the probability of the loss (instead of using a deterministic loss proportional to the size of the error) makes the scoring rule insensitive to differing risk preferences among participants^23^.

To calculate the expected value (${\rm {{EV}}}$) of a trial, we can use Eq. (1). Consider that the expected value of a trial is the sum of two products: (1) the product of the endowment $N$ and the probability of keeping the endowment and (2) the product of the endowment minus the penalty $W$ and the probability of losing the penalty from the endowment:

$${{ {EV}}}=N\left(1-{\left(I-\pi \left(Q|x\right)\right)}^{2}\right)+\left(N-W\right){\left(I-\pi \left(Q|x\right)\right)}^{2}$$

Therefore, the expected value of a trial in which the questioned gallery is the hidden gallery is

$${{{{EV}}}}_{I=1}=N\left(1-{\left(1-\pi \left(Q|x\right)\right)}^{2}\right)+\left(N-W\right){\left(1-\pi \left(Q|x\right)\right)}^{2}$$

And the expected value of a trial in which the questioned gallery is not the hidden gallery is

$${{E{V}}}_{I=0}=N\left(1-\pi {\left(Q|x\right)}^{2}\right)+\left(N-W\right)\pi {\left(Q|x\right)}^{2}$$

Since the probability that the hidden gallery is the questioned gallery is $\Pr \left(Q|x\right)$, $\Pr \left(I=1\right)=\Pr \left(Q|x\right)$ and $\Pr (I=0)=1- \Pr (Q|x)$. Therefore, the expected value of a trial after substituting the indicators for the probabilities of their respective states is

$${{{EV}}} =	 \Pr \left(Q|x\right){{{{EV}}}}_{I=1}+\left(1-\Pr \left(Q|x\right)\right){{{{EV}}}}_{I=0}\\ {{{EV}}}=	 \Pr \left(Q|x\right)\left(N\left(1-{\left(1-\pi \left(Q|x\right)\right)}^{2}\right)+\left(N-W\right){\left(1-\pi \left(Q|x\right)\right)}^{2}\right)\\ 	+\left(1-\Pr \left(Q|x\right)\right)\left(N\left(1-\pi {\left(Q|x\right)}^{2}\right)+\left(N-W\right)\pi {\left(Q|x\right)}^{2}\right)$$

which simplifies to Eq. (2).

2 $${{{EV}}}=N-W\left(\Pr \left(Q|x\right)-2\Pr \left(Q|x\right)\pi \left(Q|x\right)+\pi {\left(Q|x\right)}^{2}\right)$$

For an ideal observer who submits the exact objective posterior probability, the expected value ${{{{EV}}}}_{{{{ideal}}}}$ of an estimation trial is given by Eq. (3).

3 $${{{{EV}}}}_{{{\rm {ideal}}}}=N-W\Pr \left(Q|x\right)\left(1-\Pr \left(Q|x\right)\right)$$

Since the Museum Inference Task only accepts reported probabilities in bins (Fig. 1A), on the real task, ${p}_{{{\rm {loss}}}}$ is minimized by reporting a subjective posterior as close as possible to the objective posterior. Assuming that participants believe that their reported subjective posteriors are equal to the objective posteriors, we can calculate the subjective expected value ${{{{EV}}}}_{{{\rm {subjective}}}}$ by replacing the objective posterior probability in Eq. (3) with the subjective posterior probability $\pi \left(Q|x\right)$ (Eq. (4)).

4 $${{{{EV}}}}_{{{\rm {subjective}}}}=N-W\pi \left(Q{{{{{\rm{|}}}}}}x\right)\left(1-\pi \left(Q{{{{{\rm{|}}}}}}x\right)\right)$$

### Performance-based exclusion criteria

To ensure participant comprehension and engagement during the scan session, we assessed participants’ performance during the prescan session before we allowed them to advance to the scan session. Participants had to meet the following criteria pertaining to the Museum Inference Task to advance to the scan session: (1, comprenhension) they had to correctly answer at least 80% of the questions on a comprehension quiz on the task instructions; (2) they could miss no more than 6 percent of trials; (3, minimal sensitivity to prior probability) on trials without a sample, the Pearson correlation between reported subjective posterior and the objective posterior must have been at least 0.89 (*α* = 0.05) (this high correlation coefficient was attained by the vast majority of participants, with only 9% (4 of 44) being excluded based on this criterion alone); and (4, minimal sensitivity to objective posterior probability) the subjective posterior probability must have been significantly higher (*α* = 0.05, two-sample t-test assuming unknown and unequal variances) on trials with a high objective posterior probability (Pr(*Q* | *x*) ≥ 0.9) than on trials with a low objective posterior probability (Pr(*Q* | *x*) ≤ 0.1).

To measure participants’ intrinsic posterior-estimation strategies without extensive training, the criteria were designed to be lenient enough to respect variation in their pre-task strategies while excluding participants who disengaged from the task or who adopted strategies clearly consistent with misunderstanding the task.

### Face–Place Localizer

To localize face- and place-selective visual modules, we included a face−place functional localizer during the scan session. During the localizer task, participants viewed a picture of a face or a place on a gray background for 1 s, followed by a fixation cross for ^1^/_3_ s. Stimuli were blocked by type (face or place); each block consisted of 12 presentations of the same picture category followed by a rest period of 16^1^/_3_ s. Participants completed two runs of the Face-Place Localizer. Each run consisted of 10 blocks. The Face-Place Localizer was administered as a one-back task: participants had to right-click on a trackball if the picture on screen was the same as the previous picture while they had to left-click if the picture on screen was different from the previous picture.

### Image sets

The same image sets were used in the Museum Inference Task, Face−Place Localizer, and Museum Averaging Task. Images of faces were selected from the CNBC Faces database by Michael J. Tarr, Center for the Neural Basis of Cognition and Department of Psychology, Carnegie Mellon University, http://www.tarrlab.org, funded by NSF award 0339122, used in Righi et al. ^66^, and are available under a Creative Commons Attribution-NonCommercial-ShareAlike 3.0 Unported License. Images of places were selected from the database for Konkle et al. ^67^, available from the Computational Perception and Cognition Lab at MIT (http://olivalab.mit.edu/MM/sceneCategories.html).

### Earnings

Compensation for the prescan session was a show-up fee of $15 on top of their earnings from the payout trial (up to $30) on the prescan session. Compensation for the scan session was a show-up fee of $20 on top of their earnings from the payout trial (up to $30) on the scan session. Participants received an extra $50 for completing both sessions. Therefore, they could earn up to $145 for completing the entire study.

### Experimental session for the Museum Averaging (Control) Task

Effectively, the Museum Averaging Task session was the same as the prescan session of the Museum Inference Task but with the cover story and instructions modified so that participants were to estimate the mean of two described probabilities instead of estimating the posterior probability from a described prior probability and likelihood (Supplementary Fig. 3a, c). Participants completed the Averaging Task at a computer outside of the scanner; there was no scan session on the Museum Averaging Task.

There were 130 trials (non-catch trials and 10 catch trials), divided evenly into four runs (two “face” runs and two “place” runs, with the category alternating by run). On each non-catch trial, there were two independent wings of a gallery. Both galleries contained pictures of faces and places but in different mixtures. On a trial, the face−place mixture for the first gallery (Gallery 1) was shown as complementary percentages of faces and places, in place of the prior probabilities of the portrait and landscape galleries from the Inference Task (Fig. 1). The face−place mixture for the second gallery (Gallery 2) was shown as one randomly sampled picture from Gallery 2 along with the ratio of majority-category pictures to minority-category pictures, analogous to the majority–minority ratio and sample picture from the Inference Task (Fig. 1a). As with the Inference Task, the true sample picture was marked by an arrow to differentiate it from a decoy picture from the opposite category that appeared beside it. Then, using a slider, the participant submitted their estimate of the mean mixture across both wings with respect to the “questioned” category; the prompt was similar to that on the Inference Task except that it read, “What is the average percentage of FACES?” on face runs and “What is the average percentage of PLACES?” on place runs. Therefore, even though the Averaging Task’s solicited response was different from that of the Inference Task, the Gallery 1 and Gallery 2 probabilities were presented similarly to the prior and likelihood, respectively, on the Inference Task so that participants would have the same cognitive demand to switch the probabilities of each framing with respect to a prompt (Fig. 1a).

Analogous to the Inference Task, on catch trials, Gallery 2 was closed (i.e., the majority–minority ratio was missing and there were two decoy pictures), so the participant had to submit the Gallery 1 Probability instead of a mean. Penalties, intertrial intervals, and within-trial spatial and temporal presentation orders were the same as during the prescan session of the Inference Task. The incentivization scheme was also the same except that the binarized scoring rule directly incentivized participants to report the mean of the Gallery 1 and Gallery 2 probabilities instead of indirectly incentivizing them to report a posterior probability by proxy of a hidden gallery.

### Overview of behavioral modeling

Twenty-three participants each completed 130 trials (including catch trials) of the task, meaning that each participant was measured repeatedly. To account for this, we implemented linear mixed-effects regression to properly account for between-participant (fixed-effects) variance and within-participant (random-effects) variance, using the MATLAB function fitlme with maximum-likelihood estimation. In all mixed-effects models, we used the Satterthwaite approximation to calculate degrees of freedom, which has been shown to reduce Type 1 error compared to residual degrees of freedom^69^. All statistical tests were two-sided.

### Modeling reaction time

We used linear mixed-effects regression to model reaction time (${\rm {{RT}}}$) as a function of subjective posterior probability $\pi \left(Q|x\right)$ (Eq. (5)). Considering that slider movement and the unsigned difference between prior probability and likelihood could have affected reaction time (reasoning that the latter scenario could occur if individuals used a mental arithmetic strategy), we controlled for the absolute value of slider displacement (the distance between the initial and final slider positions, $\left|D\right|$) and the absolute difference between prior probability and likelihood ($\left|\Pr \left(Q\right)-\Pr \left(x|Q\right)\right|$). In the mixed-effects model, we used fixed-effects terms for each of these variables and included random-effects terms for each variable by participant (Eq. (5)). Results held in analyses not controlling for these covariates.

5 $${\rm {RT}} \, \sim 	 \, {\beta }_{0}+{\beta }_{{(\pi (Q|x))}^{2}}{(\pi (Q|x))}^{2}+{\beta }_{\pi (Q|x)}\pi (Q|x)+{\beta }_{|D|}|D|\\ 	 +{\beta }_{|\Pr (Q)-\Pr (x|Q)|}|\Pr (Q)-\Pr (x|Q)| +\bigg({\beta }_{0}+{\beta }_{{(\pi (Q|x))}^{2}}{(\pi (Q|x))}^{2}\\ 	 +{\beta }_{\pi (Q|x)}\pi (Q|x)+{\beta }_{|D|}|D| +{\beta }_{|\Pr (Q)-\Pr (x|Q)|}|\Pr (Q)-\Pr (x|Q)||{{{{{\rm{participant}}}}}}\bigg)$$

We also used Eq. (5) to model reaction time on the Museum Averaging Task, replacing subjective posterior probability with the reported mean and absolute difference between prior and likelihood with the absolute difference between the Gallery 1 and Gallery 2 probabilities.

### Modeling subjective posterior probability

The objective of the Museum Inference Task is to estimate the posterior probability of the questioned gallery conditional on the sample from the hidden gallery ($\Pr \left(Q|x\right)$). According to Bayes’ theorem, this posterior probability is a function of the prior probability of the questioned gallery ($\Pr \left(Q\right)$) and the likelihood of the sample conditional on the questioned gallery ($\Pr \left(x|Q\right)$) (Eq. (6)).

6 $$\Pr \left(Q|x\right)=\frac{\Pr \left(Q\right)\Pr \left(x|Q\right)}{\Pr \left(Q\right)\Pr \left(x|Q\right)+\left(1-\Pr \left(Q\right)\right)\left(1-\Pr \left(x|Q\right)\right)}$$

On each trial, the prior probabilities of the portrait and landscape galleries were explicitly stated while the likelihood was conveyed by the revealed sample picture and the sample’s evidence strength $\theta$, displayed as the ratio of majority-category to minority-category pictures (i.e., 60:40; Fig. 1a). For the purposes of the formulae, this ratio was converted into a probability (evidence strength, $\theta$) with domain $0.5 < \theta \le 1$ (e.g., 60:40 became 0.6). (However, all evidence strengths used on the task were <1). The relationship between evidence strength and likelihood $\Pr (x|Q)$ depended on the trial’s questioned gallery: $\Pr \left(x|Q\right)=\theta$ when the sample signaled the questioned gallery (i.e., when the sample was a face and the questioned gallery was the portrait gallery, or when the sample was a place and the questioned gallery was the landscape gallery), and $\Pr \left(x|Q\right)=1-\theta$ when the sample did not signal the questioned gallery.

To measure the effects of prior probability and likelihood on participants’ reported subjective posteriors, we parameterized Bayes’ theorem^13,27,47,70^. To do so, we first applied the logit transformation (Eq. (7), where $p$ is the probability to be transformed) to Bayes’ theorem to express it as a sum of logits (log odds), allowing us to model subjective posterior linearly.

7 $${{{{{\rm{logit}}}}}}\left(p\right)={{{{\mathrm{ln}}}}}\left(\frac{p}{1-p}\right),{{{{{\rm{if}}}}}}\,0 \, < \, p \, < \, 1$$

8 $${{{{{\rm{logit}}}}}}\left(\Pr \left(Q|x\right)\right)={{{{{\rm{logit}}}}}}\left(\Pr \left(Q\right)\right)+{{{{{\rm{logit}}}}}}\left(\Pr \left(x|Q\right)\right)$$

Equation (8) simply states that the logit posterior is the sum of the logit prior and the logit likelihood (log-likelihood ratio). From here, we parameterized the influence of prior and likelihood on the subjective posterior probability ($\pi \left(Q|x\right)$) using linear mixed-effects regression.

To model subjective posterior probability as a function of the objective posterior probability, we included fixed-effects terms for the intercept and objective logit posterior along with the corresponding random-effects terms by participant (Eq. (9)). To account for the potentially confounding effects of penalty ($W$) and initial slider position ($S$), we also added fixed- and random-effects terms for these effects as nuisance regressors. The parameters ${\beta }_{{{{{{\rm{logit}}}}}}\left(\Pr ({Q|x})\right)}$ and ${\beta }_{0}$ in Eq. (9) are equivalent to the probability weighting and elevation parameters, respectively, from Prospect Theory^33,34^.

9 $$\begin{array}{c}{{{{{\rm{logit}}}}}}\left(\pi \left(Q|x\right)\right) \sim {\beta }_{0}+{\beta }_{{{{{{\rm{logit}}}}}}\left(\Pr (Q{{{{{\rm{|}}}}}}x)\right)}{{{{{\rm{logit}}}}}}\left(\Pr \left(Q|x\right)\right)+{\beta }_{W}W+{\beta }_{S}S\\ +\left({\beta }_{0}+{\beta }_{{{{{{\rm{logit}}}}}}\left(\Pr (Q{{{{{\rm{|}}}}}}x)\right)}{{{{{\rm{logit}}}}}}\left(\Pr \left(Q|x\right)\right)+{\beta }_{W}W+{\beta }_{S}S|{{{{{\rm{participant}}}}}}\right)\end{array}$$

To model subjective posterior probability as a function of prior and likelihood, we included fixed- and random-effects terms for the intercept, logit prior, logit likelihood, penalty, and initial slider position (Eq. (10)).

10 $${{{{{\rm{logit}}}}}}(\pi (Q|x)) \sim 	 \,{\beta }_{0}+{\beta }_{{{{{{\rm{logit}}}}}}(\Pr (Q))}{{{{{\rm{logit}}}}}}(\Pr (Q))+{\beta }_{{{{{{\rm{logit}}}}}}(\Pr (x|Q))}{{{{{\rm{logit}}}}}}(\Pr (x|Q))\\ 	 +{\beta }_{W}W+{\beta }_{S}S +\bigg({\beta }_{0}+{\beta }_{{{{{{\rm{logit}}}}}}(\Pr (Q))}{{{{{\rm{logit}}}}}}(\Pr (Q))\\ 	+{\beta }_{{{{{{\rm{logit}}}}}}(\Pr (x|Q))}{{{{{\rm{logit}}}}}}(\Pr (x|Q))+{\beta }_{W}W+{\beta }_{S}S|{{{{{\rm{participant}}}}}}\bigg)$$

Model criterion scores for all tested models are plotted in Supplementary Fig. 2. Fixed-effects coefficients (weights) from this model are displayed in Fig. 2d and Supplementary Table 1. Because participants could only submit a subjective posterior probability between 0.02 and 0.98, inclusive, there was no risk of a nonfinite subjective logit posterior (Eq. (7)) that would make the models inestimable. A corresponding ideal observer was simulated for each participant, submitting a posterior probability estimate as close to the objective posterior probability as possible within the limitations of the accepted responses on the slider.

### Model comparison between inference and averaging behavior

To determine whether participants’ probability estimates were better explained by Bayesian inference or averaging, we applied two nonlinear models to participants’ estimates to the prescan and scan sessions of the Inference Task and to the Averaging Task: the Weighted Bayesian Model and the Mean Model. We applied these models individually to each participant so that we could compare the models’ protected exceedance probabilities from their Akaike Information Criteria (AIC) and Bayesian Information Criteria (BIC). These models were fit using the MATLAB function fitnlm with initial values of 0 for all free parameters.

The mean model (Eq. (11)) modeled probability estimates ($\hat{p}$) as a function of the mean of the prior probability and likelihood for the Inference Task or the mean of the Gallery 1 and Gallery 2 probabilities for the Averaging Task with respect to the questioned category ($\mu$); the penalty ($W$); and the initial slider position ($S$). Its free parameters were an intercept (${\beta }_{0}$) and coefficients for the penalty (${\beta }_{W}$) and initial slider position (${\beta }_{{S}}$).

11 $$\hat{p} \sim {\beta }_{0}+\mu +{\beta }_{W}W+{\beta }_{S}S$$

The Weighted Bayesian Model was based on the model of subjective logit posterior used to fit the responses to the scan session of the Inference Task in the main text (Eq. (10)) but re-expressed in probability space. We fit this model in probability space instead of logit space so that the dependent variable would be in probabilities like the Mean Model, making the AIC and BIC scores of the two models comparable for the calculation of the protected exceedance probabilities. We adopted one version of this model for the Inference Task (Eq. (12)) and the other for the Averaging Task (Eq. (13)). These versions are analogous to the Mean Model, but the Inference Task version replaces the mean with weighted prior probability ($\Pr \left(Q\right)$) and likelihood ($\Pr \left(x|Q\right)$), while the Averaging Task version replaces the mean with the product of the weighted Gallery 1 Probability ($\Pr \left({G}_{1}\right)$) if it had been a prior probability and the weighted Gallery 2 Probability ($\Pr \left({G}_{2}\right)$) if it had been a likelihood. The Inference Task version contains additional free parameters for the prior (${\beta }_{\Pr \left(Q\right)}$) and likelihood (${\beta }_{\Pr ({x|Q})}$) weights, while the Averaging Task version contains additional free parameters for the Gallery 1 (${\beta }_{\Pr \left({G}_{1}\right)}$) and Gallery 2 (${\beta }_{\Pr \left({G}_{2}\right)}$) probabilities.

12 $$\hat{p} \sim 1-\frac{1}{{\left(\frac{\Pr \left(Q\right)}{1-\Pr \left(Q\right)}\right)}^{{\beta }_{\Pr \left(Q\right)}}{\left(\frac{\Pr \left(x|Q\right)}{1-\Pr \left(x|Q\right)}\right)}^{{\beta }_{\Pr (x{{{{{\rm{|}}}}}}Q)}}\exp \left({\beta }_{0}+{\beta }_{W}W+{\beta }_{S}S\right)+1}$$

13 $$\hat{p} \sim 1-\frac{1}{{\left(\frac{\Pr \left({G}_{1}\right)}{1-\Pr \left({G}_{1}\right)}\right)}^{{\beta }_{\Pr \left({G}_{1}\right)}}{\left(\frac{\Pr \left({G}_{2}\right)}{1-\Pr \left({G}_{2}\right)}\right)}^{{\beta }_{\Pr \left({G}_{2}\right)}}\exp \left({\beta }_{0}+{\beta }_{W}W+{\beta }_{S}S\right)+1}$$

### fMRI data acquisition

Whole-brain fMRI data were acquired on a 3-T Siemens MAGNETOM Prisma scanner with a 64-channel head coil at the Magnetic Resonance Imaging Center at the Zuckerman Mind Brain Behavior Institute of Columbia University. Functional images were acquired with a T2*-weighted, two-dimensional gradient echo spiral in/out pulse sequence (repetition time (TR) = 1000 ms; echo time = 30 ms; flip angle = 52°, field of view = 230 mm; 2.4 × 2.4 × 2.4 mm voxels; 56 slices; multiband factor = 4). To reduce dropout in central frontal regions, slices were tilted by 10° forward from the AC–PC axis. During the scan session, the behavioral tasks were projected onto a mirror attached to the scanner head coil for the participant to see (Hyperion MRI Digital Projection System); participants made responses with the right hand through an MRI-compatible trackball (Current Design). fMRI data were preprocessed using fMRIPrep.

### fMRI data analysis overview

Statistical analyses were conducted using the general linear model (GLM) framework implemented in SPM12, Version 7487 (https://www.fil.ion.ucl.ac.uk/spm), convolving boxcar functions and parametric modulators within the GLM by the SPM canonical hemodynamic response function. Statistical maps from functional data were overlaid on an average of the 23 participants’ individual T1-weighted (T1w) maps normalized to Montreal Neurological Institute (MNI) space. Since scanning did not occur during the Payout Stage, fMRI activation was only measured during the Estimation Stage.

### fMRI preprocessing

Preprocessing was performed using the *fMRIPrep* pipeline, Version 1.5.0rc1^71^ (RRID:SCR_016216). fMRIPrep uses a combination of tools from well-known software packages, including FSL, ANTs, FreeSurfer, and AFNI, and is based on *Nipype* 1.2.0^72^ (RRID:SCR_002502). For more details of the pipeline, see the section corresponding to workflows in *fMRIPrep*’s documentation at (https://fmriprep.org/en/latest/workflows.html).

For anatomical data preprocessing, the T1-weighted (T1w) image was corrected for intensity nonuniformity with N4BiasFieldCorrection^73^, distributed with ANTs 2.2.0^74^ (RRID:SCR_004757). The T1w image was then skull-stripped with a *Nipype* implementation of the antsBrainExtraction.sh workflow (from ANTs), using OASIS30ANTs as the target template. Brain tissue segmentation of cerebrospinal fluid, white matter, and gray matter was performed on the brain-extracted T1w using fast^75^ (FSL 5.0.9, RRID:SCR_002823). Volume-based spatial normalization to Montreal Neurological Institute (MNI) space (MNI152NLin2009cAsym) was performed through nonlinear registration with antsRegistration (ANTs 2.2.0)^76^ (RRID:SCR_008796).

For functional data preprocessing, a skull-stripped susceptibility distortion-corrected BOLD reference was generated using a custom methodology of *fMRIPrep*. The BOLD reference was co-registered to the T1w reference using bbregister (FreeSurfer), which implements boundary-based registration using six degrees of freedom^77^. Head-motion parameters (*x*, *y*, *z*, pitch, roll, and yaw) with respect to the BOLD reference were estimated before spatiotemporal filtering using mcflirt (FSL 5.0.9)^78^. BOLD runs were slice-time corrected using 3dTshift from AFNI 20160207^79^ (RRID:SCR_005927).

### Whole-brain localization analyses for the Museum Inference Task

Functional images normalized to Montreal Neurological Institute (MNI) space were smoothed with a Gaussian kernel with a full width at half maximum (FWHM) of 5 × 5 × 5 mm before a whole-brain localization analysis was performed with a summary statistics approach. First, a voxel-wise contrast map was estimated in a first-level (participant-level) analysis for every participant from their functional image time series. All first-level GLMs for whole-brain localization of predictors used a variable-epoch model to model participants’ responses^80^: each GLM contained one boxcar function to model the decision period (the period between the beginning of the response window and the reaction time on non-catch trials that received a response (Fig. 1a; henceforth called the response boxcar) and another boxcar function to model the same period during catch trials. The response boxcar was parametrically modulated by a set of predictors that varied by localization GLM (see below). (SPM orthogonalization was turned off for all regressors, including parametric modulators^81^.) If the participant failed to respond to at least one trial during a run of the Estimation Stage, a third boxcar function was added to model the entire response window on the trials that they omitted. The localization GLMs also contained fixed-body motion-realignment regressors (*x*, *y*, *z*, pitch, roll, and yaw) and their respective first derivatives. Each participant’s contrast map was submitted to a second-level *t*-test at the group level, applying a cluster-wise correction for multiple comparisons using non-parametric permutation tests in SnPM13.1.08 (http://nisox.org/Software/SnPM13/)^63^, which have been shown to be more robust to false positives^82,83^. Permutation tests were based on a stringent cluster-forming threshold of *p* = 0.001 and considered significant at a cluster-wise familywise error rate threshold of *p* < 0.05; we used 10,000 permutations and applied variance smoothing of group-level images by a Gaussian kernel with a FWHM of 5 × 5 × 5 mm, consistent with recommendations^63,84^.

In the GLM to localize subjective logit posterior according to participants’ reports (WB-GLM 1, our main a priori GLM), the response boxcar was parametrically modulated by (1) the absolute value of the subjective logit posterior ($\left|{{{{{\rm{logit}}}}}}\left(\pi \left(Q|x\right)\right)\right|$, a representation of the subjective posterior certainty), (2) a dummy variable indicating if the subjective posterior favored the questioned gallery (yes = 1, no = −1), (3) a dummy variable indicating the side of the screen on which the sample picture appeared (sample on right = 1, sample on left = −1), (4) the penalty, (5) the subjective logit posterior of the questioned gallery (${{{{{\rm{logit}}}}}}\left(\pi \left(Q|x\right)\right)$, the product of modulators 1 and 2), (6) the subjective expected value (Eq. (4)), and (7) the initial slider position normalized to between 0 and 1.

The GLM to localize *objective* logit posterior (WB-GLM 2) had the same response boxcar and parametric modulators as WB-GLM 1 except that the objective logit posterior of the questioned gallery (${{{{{\rm{logit}}}}}}\left(\Pr \left(Q|x\right)\right)$) and the expected value according to an ideal observer (Eq. (3)) replaced the subjective logit posterior and subjective expected value, respectively.

In the GLM to localize logit prior and logit likelihood (WB-GLM 3), the response boxcar was parametrically modulated by (1) the absolute value of the logit prior ($\left|{{{{{\rm{logit}}}}}}\left(\Pr \left(Q\right)\right)\right|$, a representation of the prior certainty), (2) the absolute value of the logit likelihood ($\left|{{{{{\rm{logit}}}}}}\left(\Pr \left(x|Q\right)\right)\right|$, a representation of the likelihood certainty and evidence strength/majority−minority ratio), (3) a dummy variable indicating if the logit prior favored the questioned gallery (yes = 1, no = −1), (4) a dummy variable indicating if the logit likelihood favored the questioned gallery (yes = 1, no = –1), (5) a dummy variable indicating the side of the screen on which the sample picture appeared (sample on right = 1, sample on left = −1), (6) the penalty, (7) the logit prior of the questioned gallery (${{{{{\rm{logit}}}}}}\left(\Pr \left(Q\right)\right)$, the product of modulators 1 and 3), (8) the logit likelihood conditional on the questioned gallery (${{{{{\rm{logit}}}}}}\left(\Pr \left(x|Q\right)\right)$, the product of modulators 2 and 4), (9) the subjective expected value (Eq. (4)), and (10) the initial slider position normalized to between 0 and 1. In the exploratory conjunction analysis (Supplementary Fig. 6), we assessed the contrast between the effects of logit prior (of the questioned gallery) and logit likelihood (of the sample, conditional on the questioned gallery) by using a conjunction null, defined as regions that showed significant activation tracking both effects^85^.

We also designed a GLM (WB-GLM 4) to localize the model-fitted subjective logit posterior ($\widehat{{{{{{\rm{logit}}}}}}\left(\pi \left(Q|x\right)\right)}$) as estimated by Eq. (10). It had the same response boxcar and parametric modulators as WB-GLM 1 except the model-fitted subjective logit posterior ($|\widehat{{{{{{\rm{logit}}}}}}\left(\pi \left(Q|x\right)\right)}|$) and subjective expected value (Eq. (4), but replacing the subjective posterior probability with its model-fitted version) replaced the raw subjective logit posterior and subjective expected value, respectively.

The fact that all the trials within a run had the same questioned gallery allowed us to apply contrasts to the modulators for any logit variables (i.e., subjective logit posterior, logit prior, and logit likelihood) to create contrasts maps of these logits with respect to the questioned gallery, portrait gallery, or landscape gallery.

### fROI analyses for the Museum Inference Task

We used a functional region of interest (fROI) approach to measure average activation tracking different predictors within a cluster or region of interest^86^. We did so by defining first-level GLMs to create whole-brain contrast maps showing the response of each voxel to a particular predictor of interest. All these GLMs were fit to participants’ normalized but unsmoothed functional time series from the Museum Inference Task except for the analyses of the face- and place-selective fROIs, which were fit to functional time series that were registered to participants’ individual, unnormalized T1w images (following Doll et al. ^36^). Then, we measured the average contrast statistic of that variable within the fROI. These fROI GLMs consisted of the same boxcar functions and motion regressors as the localization GLMs but varied by the parametric modulators for the response boxcar function.

In the GLM to measure activation tracking the objective logit posterior (fROI-GLM 1), the response boxcar was parametrically modulated by (1) the objective logit posterior (${{{{{\rm{logit}}}}}}\left(\Pr \left(Q|x\right)\right)$), (2) penalty, and (3) slider displacement (the difference between the initial slider position and the slider position at the submission of the response). In the GLM testing concurrent activation for prior and likelihood (fROI-GLM 2), the response boxcar was parametrically modulated by (1) the logit prior of the questioned gallery (${{{{{\rm{logit}}}}}}\left(\Pr \left(Q\right)\right)$), (2) the logit likelihood conditional on the questioned gallery (${{{{{\rm{logit}}}}}}\left(\Pr \left(x|Q\right)\right)$), (3) penalty, and (4) slider displacement. The GLM to measure activation tracking the *subjective* logit posterior (fROI-GLM 3) had the same parametric modulators as fROI-GLM 1 except that objective logit posterior was replaced with subjective logit posterior (${{{{{\rm{logit}}}}}}\left(\pi \left(Q|x\right)\right)$).

The GLM parametric modulators were *z*-scored in all the analyses except the analysis comparing behavioral and neural probability weighting (Fig. 3f), in which we used mean-centered rather than *z*-scored predictors. This ensured that parameter estimates for neural probability weighting were comparable to the behavioral probability weights (which, in turn, were mean-centered to keep the resulting parameters commensurate with the mathematical weights from a parameterized form of Bayes’ theorem like that in Eq. (10). Because we primarily focused on group level analyses, we performed *z*-scoring and mean-centering at the group level (i.e., after pooling the data across participants). While group-level versus individual-level normalization can considerably affect the interpretation of the results^87^, in our study, the two methods produced identical outcomes. Each participant experienced the exact same set of logit priors, logit likelihoods, objective logit posteriors, and penalties (and the same number of trials) and, thus, these main regressors of interest had identical *z*-scores at the group and individual levels (all correlation coefficients *r* = 1.0). The nuisance regressor of the initial slider position was trialwise randomized, but it too was nearly identical if computed at the group or individual levels (*r* = 0.996) as were two regressors derived from it (slider displacement, *r* = 0.993 and subjective posterior, *r* = 0.971). Thus, the choice of *z*-scoring method was not a consequential concern in our task.

We also developed control fROI-GLMs to test for nonlinear encodings of subjective logit posterior within the parieto-occipital cluster. In the GLM to measure activation tracking the subjective logit posterior only on trials where the subjective posterior probability was less than 0.8, the response boxcar for trials with subjective posterior probability <0.8 was parametrically modulated by (1) the subjective logit posterior of the questioned gallery (${{{{{\rm{logit}}}}}}\left(\pi \left(Q|x\right)\right)$), (2) penalty, and (3) slider displacement (There was a separate response boxcar for trials with subjective posteriors ≥0.8 that had no parametric modulators.). In the GLM to measure activation tracking the quadratic term for subjective logit posterior, the response boxcar was parametrically modulated by (1) the subjective logit posterior of the questioned gallery (${{{{{\rm{logit}}}}}}\left(\pi \left(Q|x\right)\right)$), (2) the square of this term (${({{{{{\rm{logit}}}}}}(\pi (Q|x)))}^{2}$), (3) penalty, and (4) slider displacement.

Note that the parametric modulator for slider displacement in the fROI GLMs is different from the parametric modulator for the initial slider position in the localization voxelwise GLMs. While initial slider position was included in the latter to control for eye and hand movement, we did not include slider displacement in those GLMs because slider displacement correlated with the subjective logit posterior. In contrast, in the fROI GLMs, we sought to introduce a more stringent test to determine if the variables of interest could survive the nuisance confound.

We used mixed-effects regression to test the effect of the predictors in each fROI analysis except the analysis to test differential activation of the face- and place-selective fROIs sorted by the questioned gallery. For each mixed-effects regression test, the dependent variable was a vector of the mean contrast statistic within each relevant fROI in each participant. In analyses of only one fROI, the independent variables were dummy variables indicating the contrast map from which the corresponding mean contrast statistic was derived, with random-effects intercepts for each participant. In analyses that included more than one fROI, the independent variables were dummy variables indicating the combination of fROI and contrast identity from which the corresponding mean contrast statistic was derived, with random-effects intercepts for each participant. We used the fixed-effects coefficients from these analyses to represent the effect of each predictor of interest within the fROI. We applied the Satterthwaite approximation to calculate degrees of freedom^69^. Here and in general we used non-parametric tests (e.g., Wilcoxon signed rank test) when data was not normally distributed according to a Lilliefors test.

When testing for differential effects by sorting trials by the questioned gallery (Fig. 4; Supplementary Fig. 7d–f), we only examined logits with respect to the questioned gallery (e.g., the subjective logit posterior of the portrait gallery only during runs where the portrait gallery was the questioned gallery) to account for framing effects created by the questioned gallery. To do so, we calculated the mean contrast statistic in each participant’s face- and place-selective fROI, and we used an ANOVA to test for a significant interaction between the fROI and the contrast statistics for subjective logit posterior, logit prior, and logit likelihoods with respect to the gallery categories.

### Face–Place Localizer

We defined a GLM to localize face- and place-selective regions of occipital and temporal cortex during the Face–Place Localizer Task in each participant. Unlike the whole-brain search GLMs and the fROI GLMs used to analyze activation in the parieto-occipital cluster, the Face−Place localizer analysis was applied to functional volumes in each participant’s native brain space (without normalization). The first-level GLM consisted of two boxcar functions: one representing the period during which a face picture appeared on screen and another representing the period during which a place picture appeared on screen. Boxcar functions were convolved with the SPM canonical hemodynamic response function. Localization analysis was small volume–corrected to include only the occipital and temporal lobes in each participant. Face-selective regions were defined with the contrast Face > Place, while place-selective regions were defined with the contrast Place > Face, both at an uncorrected *p*-value threshold of 0.001 at the individual level.

### Reporting summary

Further information on research design is available in the Nature Portfolio Reporting Summary linked to this article.

### Supplementary information

Supplementary Information

Description of Additional Supplementary Files

Supplementary Data 1

Reporting Summary

## Data Availability

Experimental data, including those used to generate Figs. 2–4, are available at Open Science Framework (https://osf.io/3vdut/?view_only=d89742283a84454681d59236c1e8b3b8)^88^.

## Code availability

The code used to analyze the data, including those in Figs. 2–4, is available at Open Science Framework (https://osf.io/3vdut/?view_only=d89742283a84454681d59236c1e8b3b8)^88^.
