# Supplementary material for: The parieto-occipital cortex is a candidate neural substrate for the human ability to approximate Bayesian inference
Source: Commun Biol. 2024 Feb 9;7:165. doi: 10.1038/s42003-024-05821-6 (PMC10858241; doi:10.1038/s42003-024-05821-6)
Supplement: Supplementary file 1 — Supplementary Information [file 42003_2024_5821_MOESM1_ESM.pdf]

Supplementary Information for
The parieto-occipital cortex is a candidate neural substrate for the human ability to approximate Bayesian inference

Nicholas M. Singletary, Jacqueline Gottlieb, and Guillermo Horga

Corresponding Authors: Nicholas M. Singletary, Jacqueline Gottlieb, and Guillermo Horga
Email: ns3068@cumc.columbia.edu, jg2141@columbia.edu, and guillermo.horga@nyspi.columbia.edu

Supplementary Note 1: Results from the Museum Averaging Task
To evaluate the possibility that participants' responses to the Museum Inference Task are explained by averaging prior probability and likelihood instead of Bayesian Inference, we developed the Museum Averaging Task, a modification of the Inference Task on which participants were asked to estimate the mean of two probabilities—the average of the probabilities of a questioned picture category between two galleries, 1 and 2—instead of the posterior probability. Then, we compared participants' responses on the Museum Averaging Task to their responses on the prescan and scan sessions of the Museum Inference Task. The trial parameters on the Averaging Task were effectively the same as those of the prescan session of the Inference Task except that the prior probability and likelihood on the Inference Task respectively became the Gallery 1 and Gallery 2 probabilities on the Averaging Task (**Supplementary Figure 3a,c**).

To facilitate comparison of behavior between the Inference and Averaging tasks, we split non-catch trials into two categories: critical trials and non-critical trials. On critical trials, the probabilities on the trial (prior and likelihood with respect to the questioned gallery on the Inference Task; Gallery 1 and 2 probabilities with respect to the questioned picture category on the Averaging Task) are both greater than 0.5, or both less than 0.5. On all other non-catch trials (non-critical trials), the trial probabilities did not meet this criterion. Probability estimates on the critical trials are most distinguishable between an inference and an averaging strategy because, on these trials, an estimate reflecting Bayesian inference should be more extreme (farther from 0.5) than both probabilities because the posterior probability would be more extreme than both the prior probability and likelihood in this case per Bayes' theorem (**Equation 6**). Meanwhile, by definition, a probability estimate reflecting averaging should be between the two probabilities given on a trial.

First, we compared the proportion of critical trials from the Inference and Averaging tasks on which the response was more extreme than the trial's given probabilities. On 45% (630 of 1,394) of completed critical trials during the prescan session and 34% (436 of 1,264) of completed critical trials during the scan session of the Inference Task, the subjective posterior probabilities were more extreme than the trial's prior probability and likelihood. In contrast, on the Averaging Task, the reported mean of the Gallery 1 and Gallery 2 probabilities was more extreme than the individual gallery probabilities on only 4% (43 of 1,153) of completed critical trials. This discrepancy suggests that, indeed, participants' strategies on the Inference Task were more aligned with Bayesian inference while their strategies on the Averaging Task were more aligned with averaging.

Second, we used a model-free analysis through linear mixed-effects regression to examine whether the difference between participants' reports and the mean of the trial probabilities is positively correlated with the difference between the objective posterior based on the trial probabilities and the mean of the trial probabilities. We predicted that if participants were mainly averaging, these differences would not be correlated because the difference between the report and the mean would be centered around 0 regardless of the difference between the objective posterior and the mean; if participants were mainly doing Bayesian inference, the differences should instead be positively correlated. Indeed, on critical trials, these differences were positively correlated during both the prescan (fixed-effect/group-effect regression slope from mixed-effects model: 0.34, N = 23 participants, $T(23.01) = 4.49$, SE = 0.07, $p < 0.001$; **Supplementary Figure 3d**) and scan session (fixed-effects regression slope: 0.19, N = 23 participants, $T(23.00) = 2.15$, SE = 0.09, $p = 0.042$, degrees of freedom calculated with Satterthwaite approximation, see **Behavioral Modeling** in section of **Methods**; **Supplementary Figure 3e**) of the Inference Task, but they were negatively correlated during the Averaging Task (fixed-effects regression slope: −0.14, N = 23 participants, $T(19.02) = -2.45$, SE = 0.06, p = 0.024; **Supplementary Figure 3f**), constituting further evidence that participants were averaging during the Averaging Task and approximating Bayesian inference during the Inference Task.

Third, we compared a model of participants' responses as the mean of prior and likelihood (**Equation 11**) to a parameterized (weighted) Bayesian model (**Equation 12**, **Equation 13**). According to protected exceedance probabilities based on AIC and BIC, the Weighted Bayesian Model outperformed the Mean Model during both sessions of the Inference Task

(**Supplementary Figure 3g–h**) while the Mean Model outperformed the Weighted Bayesian Model during the Averaging Task (**Supplementary Figure 3i**), further indicating that participants approximated Bayesian inference on the Inference Task and averaged on the Averaging Task.

Finally, inspired by theory and empirical findings that decision time decreases with evidence[1,2], we compared the relationship between reaction time and subjective posterior probability between the Inference and Averaging tasks. We predicted that when people were using approximate Bayesian inference, reaction time would peak at subjective posteriors close to 0.5 where there was the least evidence and decline as subjective posterior diverged from this value. However, if they were averaging, there would be no reason for such a relationship. Confirming our hypothesis, on both sessions of the Inference Task, there was a negative quadratic relationship between reaction time and subjective posterior probability after controlling for the absolute value of slider displacement and the absolute difference between the trial probabilities (coefficient on prescan session: −9.51, $N = 23$ participants, $T(26.19) = -11.59$, SE = 0.820, $p < 0.001$; coefficient on scan session: −5.88, $N = 23$ participants, $T(27.06) = -7.24$, SE = 0.81, $p < 0.001$; relationships with residual reaction time in **Supplementary Figure 3j–k** for visualization only); however, there was no significant quadratic effect on the Averaging Task (coefficient: −1.80, $N = 23$ participants, $T(26.43) = -1.56$, SE = 1.15, $p = 0.131$; relationship with residual reaction time in **Supplementary Figure 3l** for visualization only).

These findings constitute converging evidence that participants relied on Bayesian inference (and not averaging) on the Museum Inference Task, and that averaging behavior is distinguishable from inference behavior.

Supplementary Note 2: Effects of Logit Prior, Logit Likelihood, and Intended Final Slider Position in the Parieto-Occipital Cluster

We devised an analysis to test if, because we identified the parieto-occipital cluster based on its encoding of the logit posterior, this introduced a statistical bias to detect effects of the logit prior and logit likelihood. The analysis capitalized on the fact that, because the logit posterior is the sum of the logit prior and logit likelihood, it remains constant if we simply interchange the labels of the latter two terms. For example, the logit posterior would be 1.2 whether logit prior = 0.4 and logit likelihood = 0.8 or the labels were swapped, i.e., logit prior = 0.8 and logit likelihood = 0.4. We thus devised a permutation analysis in which we randomized the two labels and produced a null distribution showing how the activation in the parieto-occipital cluster would covary with the logit prior and logit likelihood if it merely encoded the subjective posterior without truly tracking these terms.

To generate the null distribution, we performed 1,000 permutations in which we interchanged the priors and likelihood labels of a randomly selected 50% of trials in each session and recomputed the GLM parameters for the logit prior and logit likelihood. This procedure ensured that we truly randomized rather than simply interchanged the labels (as would have happened if we switched 100% of the labels in each session) and, most crucially, ensured that we kept the logit posterior constant. Thus, the null distributions (gray in **Supplementary Figure 5**) indicate the scaling of activity that would be expected in the parieto-occipital cluster only from the cluster's encoding of the subjective posterior (or, equivalently, the final slider position) without information of the true logit prior and logit likelihood values.

The original observed GLM parameter estimates for logit prior and logit likelihood (**Figure 3d**) were greater than the entirety of the null distributions (*p*-values of 0). Moreover, the null distributions maintained similar range and shape when we restricted the permutation to a subset of iterations with very similar correlations between the logit posterior and, respectively, the logit prior and logit likelihood, as the original, non-shuffled session. The observed parameter estimates were much greater than the null distributions produced by this subset as well. Thus, the parieto-occipital cluster shows *bona fide* responses to the logit prior and logit likelihoods that would not be expected to occur based merely on its encoding of the final slider position or the subjective logit posterior.

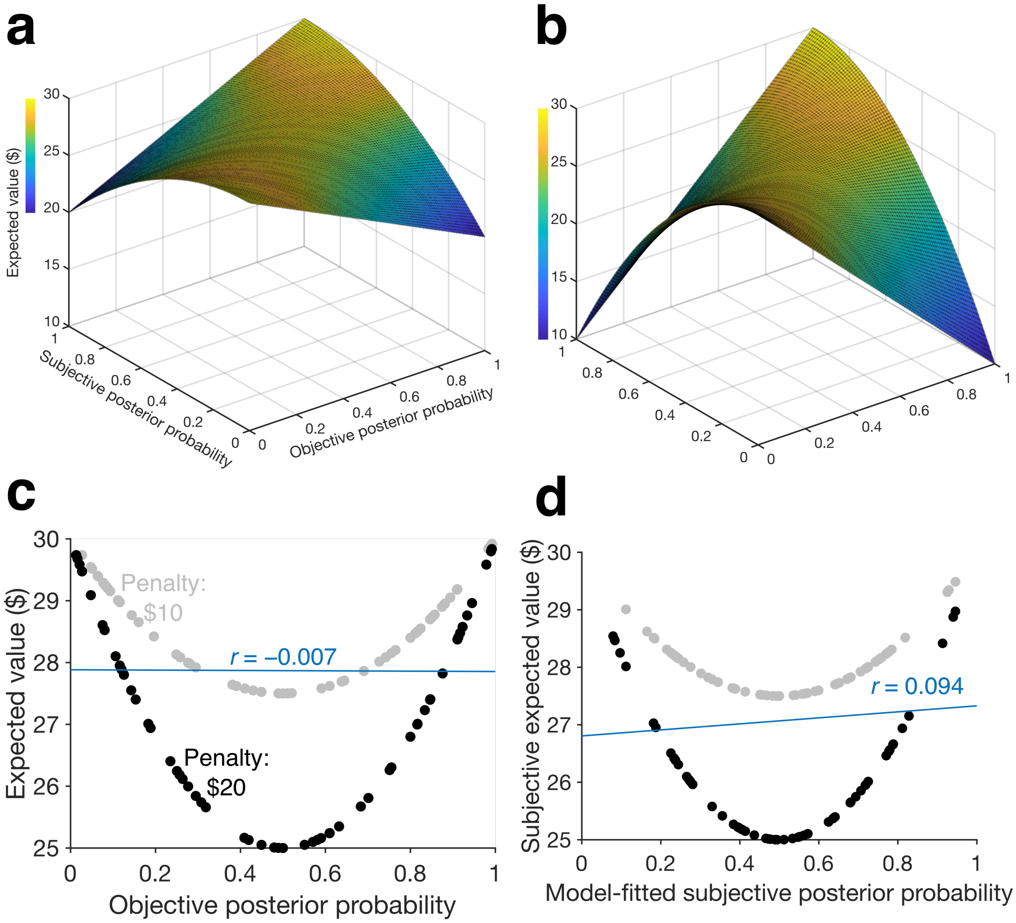

Supplementary Figure 1. Our incentivization method (**Methods**) incentivizes accuracy without linearly confounding expected value (EV, **Equation 2**) and posterior probability.

a Expected value of a trial in the $10 penalty condition over the entire space of possible subjective (submitted) and objective posterior probabilities. EV is maximized in the yellow diagonal region where subjective posterior probability is closest to objective posterior probability.

b Same as A, but for the $20 penalty condition. Note that EV is markedly lower than in the $10 condition, especially when the subjective posterior is farthest from the objective posterior.

c Duplicate of **Figure 1c**. When the subjective posterior probability is equal to the objective posterior probability (along the diagonals of the planes in A and B), there is a U-shaped relationship between objective posterior probability, but only a negligible linear correlation between posterior probability and EV across all non-catch trials (Pearson correlation: −0.001, p = 0.94, N = 120). EV declines with penalty. Gray circles represent trials with a $10 penalty. Black circles represent trials with a $20 penalty. The blue line is the least-squares line across all trials.

d There is a very low correlation between the model-fitted subjective posterior probability (**Equation 10**: model-predicted subjective posterior probability calculated using the fixed-effects—i.e., group level—regression weights) and subjective expected value (**Equation 4**) across all non-catch trials (Pearson correlation: 0.094, p = 0.09, p = 0.31, N = 120), assuming that participants sought to be as accurate as possible and believed themselves to be accurate. Uses the color scheme from **c**.

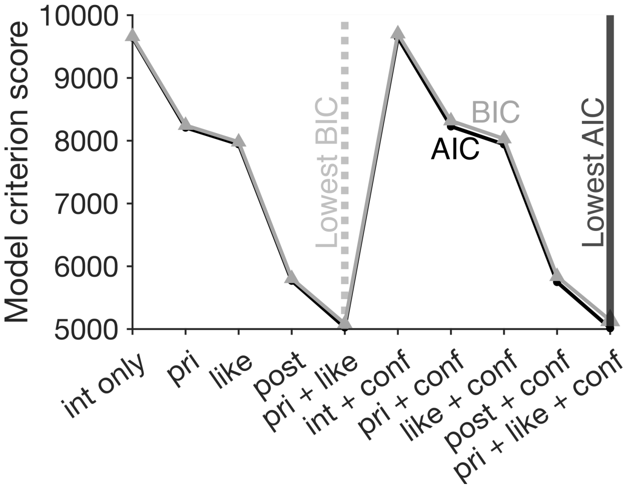

Supplementary Figure 2. Akaike Information Criterion (AIC, black circles) and Bayesian Information Criterion (BIC, gray triangles) scores for the various models of subjective logit posterior of the questioned gallery suggest that including separate regressors for logit prior and logit likelihood improves model accuracy compared to modeling subjective logit posterior only as a function of individual variables. Compares mixed-effects regression models that contain fixed- and random-effects terms for every regressor.

Regressor names:
"int only": intercept only
"pri": logit prior of questioned gallery
"like": logit likelihood of sample conditional on questioned gallery
"post": objective logit posterior of questioned gallery
"conf": penalty and initial slider position

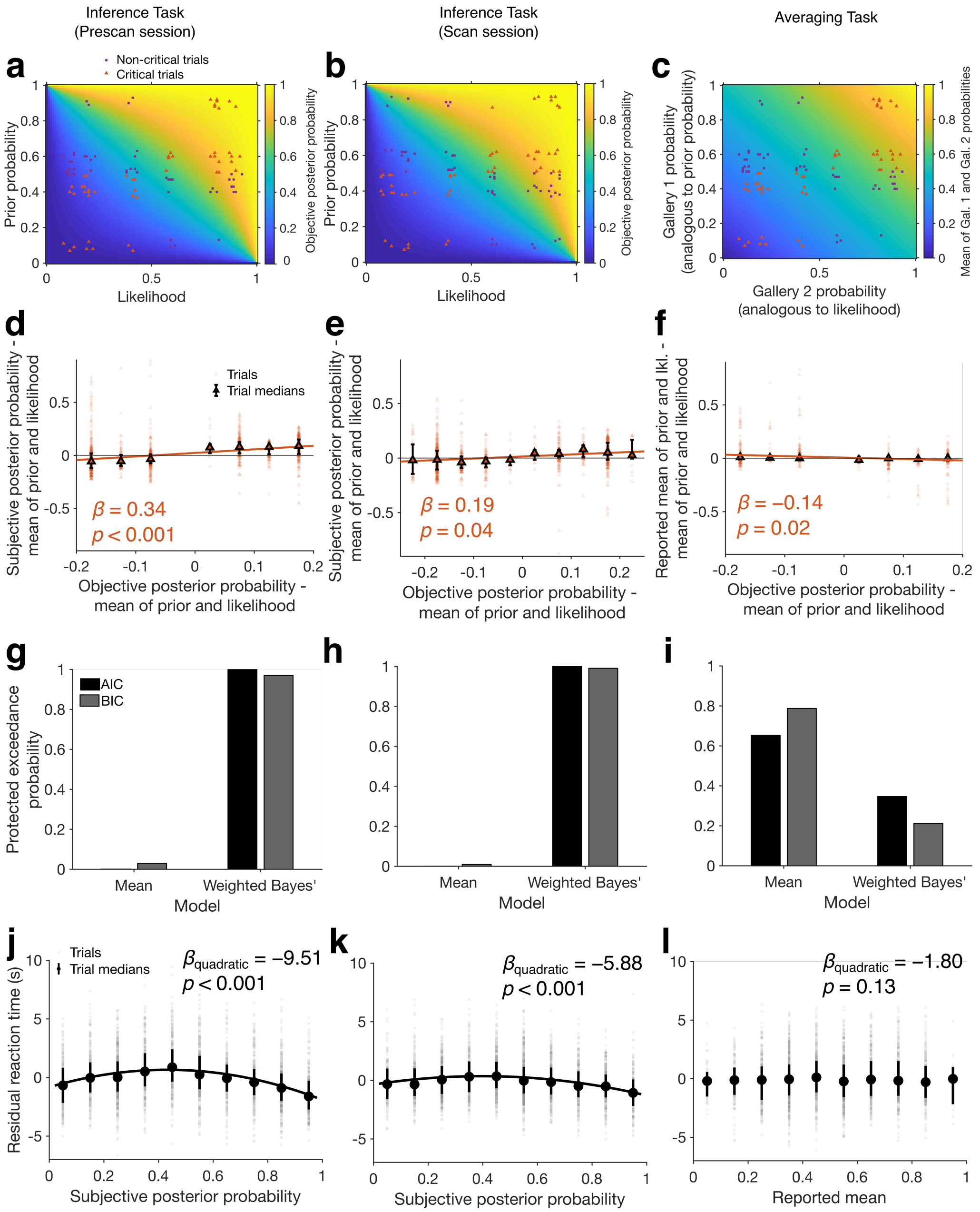

Supplementary Figure 3. Averaging, a possible mental arithmetic heuristic for Bayesian inference, does not explain participants' behavior on the Museum Inference Task as well as our Bayesian inference model does, as suggested by differences in response patterns and reaction time between two sessions of the Museum Inference Task (*N* = 23, prescan session: left column; scan session: center column) and the Museum Averaging Task (right column, *N* = 19).

a–b The objective posterior probability of the questioned gallery conditional on the sample picture (colored grid) as a function of the prior probability of the questioned gallery (*y*-axis) and the likelihood of the sample conditional on the questioned gallery (*x*-axis), with points at prior-likelihood combinations that were used on non-catch trials during the prescan (**a**) and scan (**b**, corresponds to **Figure 1b**) sessions of the Museum Inference Task. Orange triangles represent critical trials, trials on which the prior and likelihood are both greater than 0.5, or both less than

0.5. All other trials (non-critical trials) are represented by purple squares. Responses to critical trials should be the most distinguishable between the Inference and Averaging tasks because, on these trials, a response reflecting Bayesian inference should be more extreme (farther from 0.5) than both the prior and likelihood whereas a response reflecting averaging should be between the prior and likelihood.

c The mean of the probability of galleries 1 and 2 (colored grid) as a function of the Gallery 1 probability (*y*-axis) and the Gallery 2 probability (*x*-axis), with points at gallery probability combinations that were used on non-catch trials during the Museum Averaging Task. Orange trials represent critical trials while purple squares represent all other trials. Note that the Museum Averaging Task has the same parameters as the prescan session of the Museum Inference Task—the Gallery 1 probability corresponds to the prior probability whereas the Gallery 2 probability corresponds to the likelihood.

d–f: During critical trials from both sessions of the Museum Inference Task (**d–e**), the difference between participants' responses (the subjective posterior probability) and the mean of prior probability and likelihood is positively associated with the difference between the objective posterior probability and the mean of prior and likelihood, indicating that participants' responses were more driven by approximating the posterior probability than by averaging prior and likelihood. The relationship was negative during the Averaging Task (**f**).

g–i: We used protected exceedance probability (PXP) to compare two models' abilities to explain participants responses on all non-catch trials on the Inference and Averaging tasks: a "Weighted Bayes'" model (**Equation 12**, **Equation 13**) based on the Bayesian inference model used in the main text and a "Mean Model" that modeled responses as a function of the mean of prior and likelihood (**Equation 11**). PXP is the probability that a model exceeds all other models in the comparison set while protecting against the risk that the differences in model evidence occurred by chance, and it can be calculated from Akaike Information Criterion (AIC, black) and Bayesian Information Criterion (BIC, gray) scores. On both sessions of the Inference Task, the PXP of the Weighted Bayesian Model was higher than that of the Mean Model, while the converse was true on the Averaging Task, suggesting that Bayesian inference (estimating the posterior probability) better explained behavior during the Inference Task while averaging better explained behavior during the Averaging Task.

j–l: On both sessions of the Inference Task (**j–k**), participants' reports ("subjective posterior probability") had a significant negative quadratic effect on reaction time, with reaction times peaking at reports around 0.5 and falling as reports approached 0 or 1, after controlling for the absolute value of slider displacement and the absolute difference between prior and likelihood. (The black curves in **j–k** are the least-squares lines for the individual trials.) However, on the Averaging Task (**l**), participants' reports ("reported mean") were relatively flat and had no significant quadratic effect on reaction time after controlling for these nuisance variables. Points represent median reaction time after regressing out effects of slider displacement and absolute difference binned by subjective posterior probability. Error bars represent the interquartile range. However, these plots are for visualization only and were not used to test for effects. Analysis included all non-catch trials.

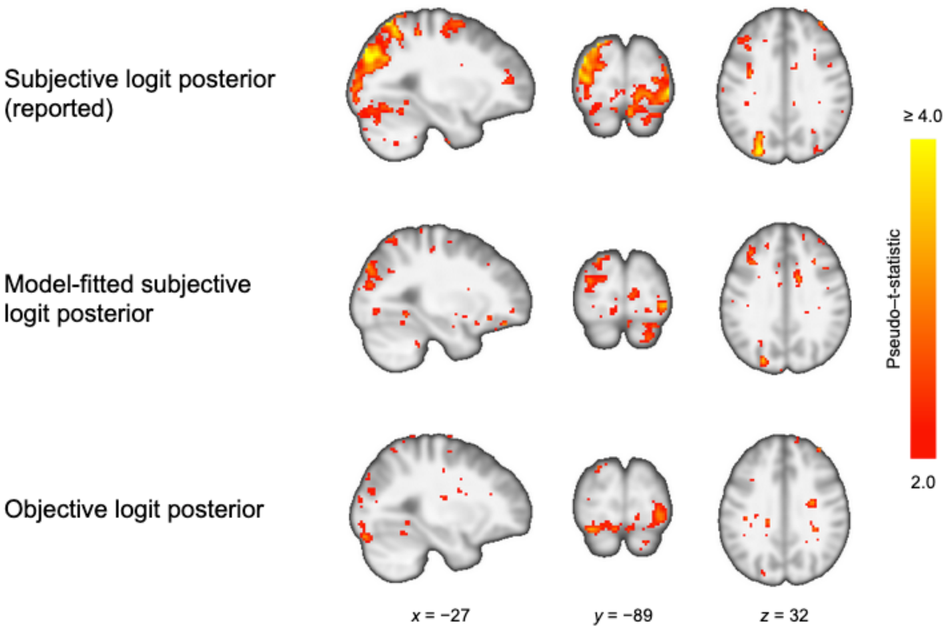

Supplementary Figure 4. Activation tracking the subjective logit posterior of the questioned gallery according to the participants' (*N* = 23) reports (top row, corresponds to **Figure 3a–b**, WB-GLM 1); model-fitted subjective logit posterior according to **Equation 10**, the same model used to yield the regression weights in **Figure 2d**; and the objective logit posterior according to Bayes' theorem (bottom row, WB-GLM 2). All activation here is displayed at a lenient threshold for visualization purposes to allow comparison across models (uncorrected t-score ≥ 2). However, BOLD activation tracking the model-fitted subjective logit posterior and the objective logit posterior do not meet the significance threshold used for the main text.

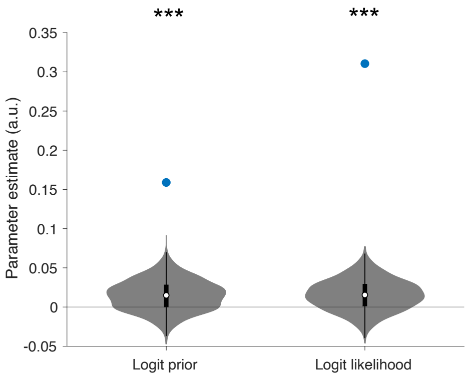

Supplementary Figure 5. Permutation test of the validity of logit prior and logit posterior GLM parameter estimates. The violin plots (gray) and the box plots on top of them (black and white) show the null distributions for the GLM parameter estimates after randomizing the logit likelihood and logit prior labels while holding the logit posterior constant. In each box plot, the white circle represents the median of the null distribution, the box represents the interquartile range, and the whiskers extend to the parameter estimates within 1.5 times the interquartile range of the first and third quartiles. The blue circles show the observed parameter estimates for logit prior and logit likelihood from the original, non-shuffled data. The asterisks indicate $p < 0.001$. The observed parameter estimates are greater than the entirety of their corresponding null distributions, indicating that the observed estimates are extremely unlikely to have been epiphenomena of the cluster's responses to the logit posterior (or, equivalently, the final slider position). This figure was created in part by using code by Bechtold[3].

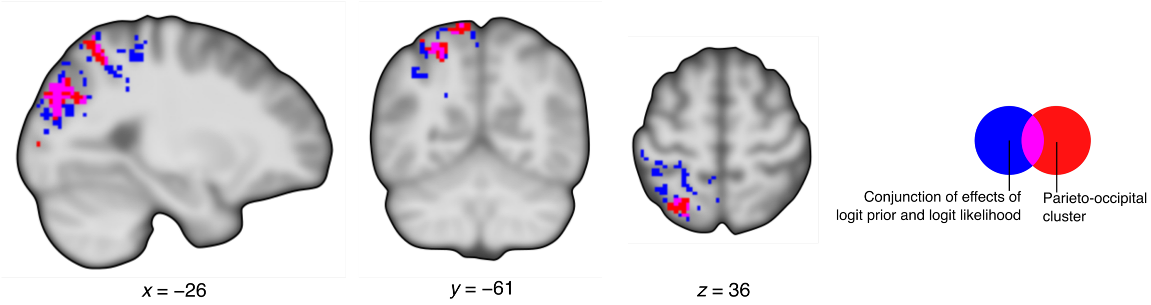

Supplementary Figure 6. A significant cluster in the left posterior parietal and anterolateral occipital cortices showed a conjunction of effects of logit prior and logit likelihood (blue). This cluster overlaps substantially with the parieto-occipital cluster tracking subjective logit posterior (red) in the superior parietal lobule and the intraparietal sulcus (overlap: purple). The conjunction cluster was created by performing a conjunction test in every participant ($N = 23$) and performing a second-level non-parametric permutation test across all the participant-level conjunction maps. Cluster-forming height threshold: $p < 0.001$. Threshold for cluster-wise family-wise error-rate correction for multiple comparisons: $p < 0.05$, with an additional cluster-extent threshold of 400 voxels for visualization purposes. MNI coordinates of slices: −26, −61, 36.

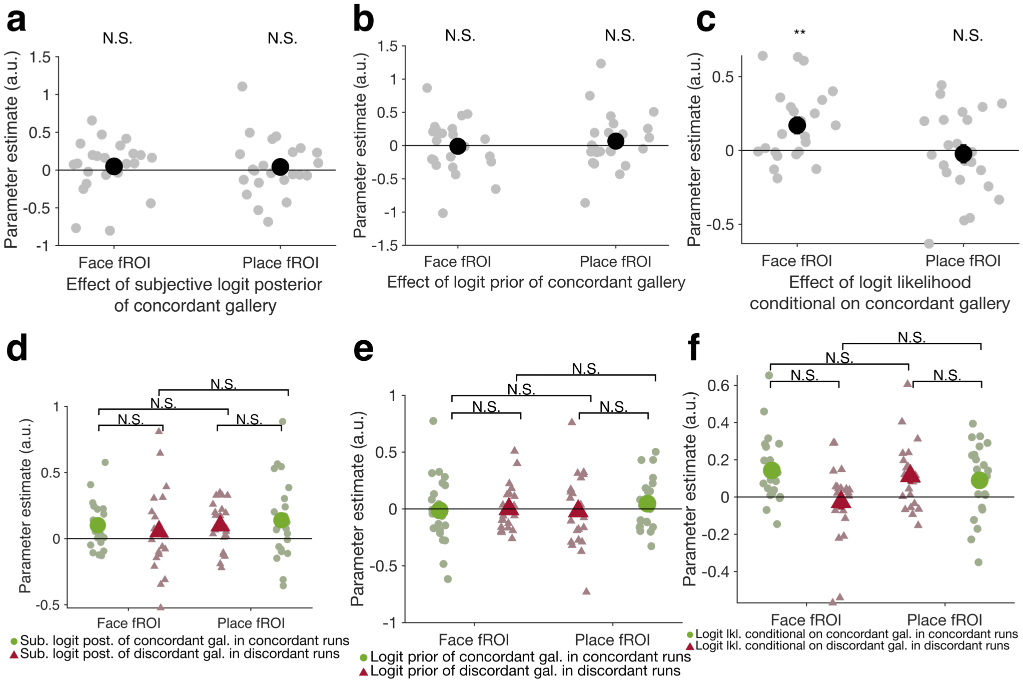

Supplementary Figure 7. Face- and place-selective functional regions of interest (fROIs) show no consistent trend of probabilistic category-concordant activation.

a–c In an analysis that included all trials, face- and place-selective fROIs were not significantly activated by the subjective logit posterior (**a**, duplication of **Figure 4b**) or logit prior (**b**) of their concordant galleries (i.e., the galleries corresponding to the “preferred” stimuli of that region: portrait gallery corresponding to face fROI and landscape gallery corresponding to place fROI). While the face fROI does show a positive effect of the logit likelihood conditional on the portrait gallery, the place fROI does not show a significant effect of the logit likelihood conditional on the landscape gallery (**c**). Because there were only two options (portrait gallery or landscape gallery), the probabilities of the two galleries are complementary within each analysis. Group-level statistics in black while participant-level statistics in gray. N.S.: “not significant.” **: $0.01 < p \leq 0.001$.

d–f After dividing trials by their questioned galleries (portrait or landscape), neither fROI showed preferential activation by the posterior probability of its concordant gallery and neither posterior probability had a higher parameter estimate in its concordant fMRI (**d**). The same applies to prior (**e**) and likelihood (**f**). Group-level statistics in saturated colors while participant-level statistics in pastel colors. Statistics for concordant galleries are green circles while statistics for discordant galleries are red triangles. N.S.: “not significant”

Error bars represent standard errors. Across 23 participants.

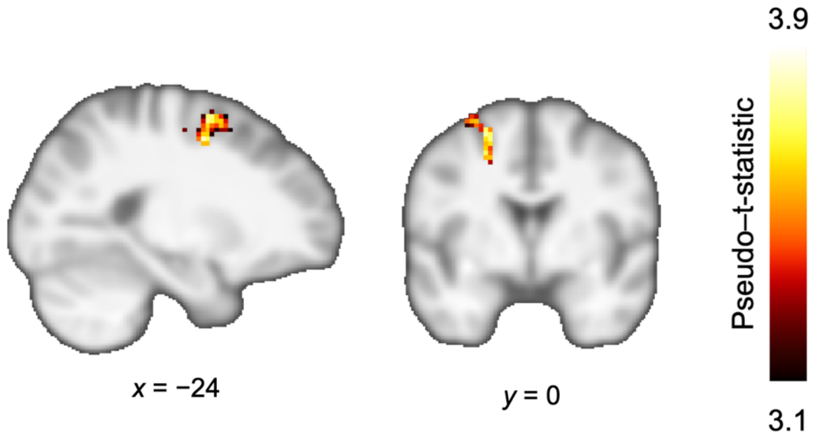

Supplementary Figure 8. One cluster in the left premotor cortex shows activation tracking subjective logit posterior of the portrait gallery but only during portrait runs. Cluster-thresholded ($p < 0.05$, corrected for familywise error rate by permutation test) with cluster-defining height threshold of 0.001. Across 23 participants.

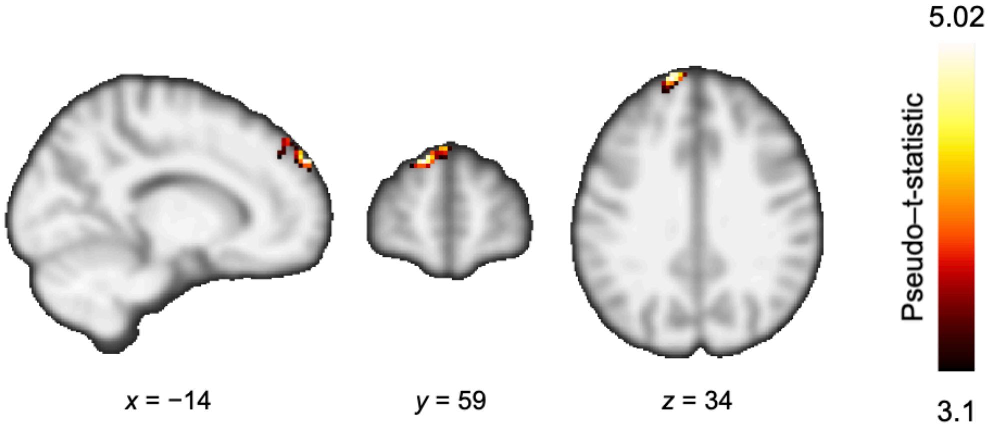

Supplementary Figure 9. One cluster in the left frontal pole shows activation tracking logit prior of the portrait gallery during all runs. Cluster-thresholded ($p < 0.05$, corrected for familywise error rate by permutation test) with cluster-defining height threshold of 0.001. Across 23 participants.

Supplementary Table 1. Fixed-effects regression weights from the mixed-effects model of subjective logit posterior as a function of logit prior, logit likelihood, and nuisance regressors (**Equation 10**). Corresponds **Figure 2d**. Z: statistics from regression with z-scored predictors. M: statistics from regression with mean-centered (but not z-scored) predictors. *DF*: Degree of freedom (from Satterthwaite approximation[4]). *p*: *p*-value. Across 23 participants.

Regressor	Regression weight		Standard error		*T*-statistic	DF		*p*	
	Z	M	Z	M		Z	M	Z	M
Intercept	0.073	0.073	0.028	0.028	2.594	22.979	23.817	0.010	0.016
Logit prior	0.789	0.661	0.056	0.047	14.172	22.991	22.994	< 0.001	< 0.001
Logit likelihood	0.804	0.502	0.078	0.049	10.302	22.999	22.808	< 0.001	< 0.001
Inaccuracy penalty	0.040	0.008	0.012	0.002	3.201	68.351	67.16	0.001	0.002
Initial slider position	0.035	6.4×10^{-5}	0.013	2.4×10^{-5}	2.659	40.123	35.957	0.008	0.012

Supplementary Table 2. Peak voxels from the parieto-occipital cluster (467 voxels) that significantly tracked subjective logit posterior (**Figure 3a**). Anatomical regions were assigned by the JuBrain/SPM Anatomy Toolbox 3.0[5–7] and the Oxford-Harvard Atlas[8]. Cortical lobes were assigned by the AAL3 Atlas[9].

Pseudo–*t*-statistic	MNI Coordinates			Anatomical region		
	x	*y*	*z*	JuBrain	Oxford/Harvard Atlas	Cortical lobe
5.07	−19	−67	49	7A (SPL)	Lateral Occipital Cortex, superior division	Parietal
4.70	−17	−58	73	N/A	Superior Parietal Lobule	Parietal
4.53	−24	−65	66	7A (SPL)	Lateral Occipital Cortex, superior division	Parietal
4.50	−27	−84	32	hIP4 (IPS)	Lateral Occipital Cortex, superior division	Occipital
4.12	−36	−89	25	hOc4lp	Lateral Occipital Cortex, superior division	Occipital
4.12	−34	−65	61	7A (SPL)	Lateral Occipital Cortex, superior division	Parietal
4.03	−24	−60	59	7A (SPL)	Lateral Occipital Cortex, superior division	Parietal
3.98	−34	−94	18	hOc4lp	Occipital Pole	N/A
3.97	−27	−77	35	hIP5 (IPS)	Lateral Occipital Cortex, superior division	Occipital
3.94	−36	−94	6	hOc4lp	Occipital Pole	Occipital

Supplementary Table 3. Coverage of parieto-occipital cluster (**Figure 3a**) by functional parcels identified by Glasser et al.[10]. Over 70% of the cluster lies within parietal regions. Percentages do not sum to exactly 100% because they were rounded. The shading was included to aid in the visual separation of the rows for each lobe.

*Percentage of the voxels assigned to a cluster.

Lobe	Parcel index	Area name	Area description	Percentage of cluster*	
				In parcel	In lobe
Parietal	17	IPS1	IntraParietal Sulcus Area 1	7.5	73.3
	46	7Pl	Lateral Area 7P	4.2	
	47	7PC	Area 7PC	0.5	
	48	LIPv	Area Lateral IntraParietal ventral	4.7	
	49	VIP	Ventral IntraParietal Complex	14.6	
	50	MIP	Medial IntraParietal Area	6.6	
	143	PGp	Area PGp	10.8	
	145	IP1	Area IntraParietal 1	10.8	
	146	IP0	Area IntraParietal 0	7.0	
	151	PGs	Area PGs	6.6	
Parieto-occipital sulcus	16	V7	Seventh Visual Area	2.8	7.5
	152	V6A	Area V6A	4.7	
Occipital	6	V4	Fourth Visual Area	8.0	19.3
	158	V3CD	Area V3CD	11.3	

Supplementary Table 4. Parameter estimates of activation of the parieto-occipital cluster (**Figure 3a**) for logit prior, logit likelihood, and nuisance regressors. Corresponds to statistics in **Figure 3c**. *DF*: Degrees of freedom (from Satterthwaite approximation[4]). *p*: *p*-value. Across 23 participants.

Regressor	Parameter estimate	Standard error	T-statistic	DF	*p*
Logit prior	0.159	0.062	2.547	92.000	0.013
Logit likelihood	0.311	0.062	4.977	92.000	< 0.001
Inaccuracy penalty	−0.057	0.062	−0.921	92.000	0.360
Slider displacement	0.040	0.062	0.635	92.000	0.527

Supplementary Table 5. Peak voxels from the four largest significant clusters exhibiting a conjunction of effects of logit prior and logit likelihood (cluster-level family-wise error rate threshold for multiple comparisons correction: 0.05). The largest cluster partially overlaps with the parieto-occipital cluster tracking subjective logit posterior and is shown in **Supplementary Figure 6**. Anatomical regions were assigned by the JuBrain/SPM Anatomy Toolbox 3.0[5–7] and the Oxford-Harvard Atlas[8]. The shading was included to aid in the visual separation between the rows for each cluster.

Cluster	Size (vox.)	Pseudo–*t*-statistic	MNI Coordinates			Anatomical region	
			x	*y*	*z*	Oxford-Harvard	JuBrain
Left posterior parietal cortex/ anterolateral occipital cortex	873	6.25	−24	−82	32	Lateral Occipital Cortex, superior division	hIP7 (IPS)
		6.05	−22	−60	30	Precuneous Cortex	N/A
		5.71	−27	−84	37	Lateral Occipital Cortex, superior division	hIP4 (IPS)
		5.50	−36	−34	42	Postcentral Gyrus	3a
		5.46	−27	−53	49	Superior Parietal Lobule	hIP3 (IPS)
		5.35	−27	−82	20	Lateral Occipital Cortex, superior division	hIP7 (IPS)
		5.30	−3	−55	71	Precuneous Cortex	N/A
		5.18	−36	−39	59	Postcentral Gyrus	2
		5.15	−27	−39	54	Postcentral Gyrus	5L (SPL)
		5.09	−43	−79	13	Lateral Occipital Cortex, superior division	hOc4la
Left posterior inferior temporal lobe/ fusiform/ inferior occipital cortex	864	6.24	−15	−96	−18	Occipital Pole	hOc3v [V3v]
		6.14	−48	−48	−23	Inferior Temporal Gyrus, temporooccipital part	FG4
		5.82	−24	−89	−18	Occipital Fusiform Gyrus	hOc4v [V4(v)]
		5.68	−22	−53	6	Precuneous Cortex	N/A
		5.65	−24	−51	−16	Temporal Occipital Fusiform Cortex	FG3
		5.42	−31	−82	−11	Occipital Fusiform Gyrus	hOc4v [V4(v)]
		5.37	−29	−67	−20	Cerebellum Left VI	FG1
		5.28	−48	−63	8	Middle Temporal Gyrus, temporooccipital part	N/A
		5.11	−41	−72	4	Lateral Occipital Cortex, inferior division	hOc5 [V5/MT]
		5.07	−29	−48	−25	Cerebellum Left VI	N/A
Right fusiform/ inferior occipital cortex	812	5.58	36	−94	8	Occipital Pole	hOc3v [V3v]
		5.47	14	−58	−6	Lingual Gyrus	hOc2 [V2]
		5.43	43	−60	−20	Temporal Occipital Fusiform Cortex	FG2
		5.40	38	−39	−28	Temporal Fusiform Cortex, posterior division	FG4
		5.24	5	−72	−1	Lingual Gyrus	hOc2 [V2]
		5.14	9	−63	−6	Lingual Gyrus	N/A
		5.03	9	−77	1	Lingual Gyrus	hOc1 [V1]
		4.99	12	−72	−6	Lingual Gyrus	hOc3v [V3v]
		4.96	33	−31	−18	Temporal Fusiform Cortex, posterior division	CA1 (Hippocampus)
		4.90	19	−34	−23	Cerebellum Right I-IV	N/A
Right frontal pole/ orbitofrontal cortex	470	5.46	19	19	−16	Frontal Orbital Cortex	Fo3
		5.29	7	19	4	Right Caudate	N/A
		5.17	19	55	−6	Frontal Pole	Fp1
		5.16	43	41	11	Frontal Pole	45
		5.14	43	48	6	Frontal Pole	N/A
		5.14	38	52	−4	Frontal Pole	N/A
		5.07	26	55	−16	Frontal Pole	Fo3
		5.06	9	43	6	Cingulate Gyrus, anterior division	p24c
		4.98	36	38	−16	Frontal Pole	Fo3
		4.97	48	31	−11	Frontal Orbital Cortex	45

Supplementary Table 6. Fixed-effects parameter estimates for activation of face and place functional regions of interest (fROIs) by the subjective logit posterior of the gallery signaled by their preferred stimuli. Corresponds to **Figure 4b** and **Supplementary Figure 7a**. *DF*: Degrees of freedom (from Satterthwaite approximation[4]). *p*: *p*-value. Across 23 participants. The shading was included to aid in the visual separation between the rows for each fROI.

fROI	Regressor	Parameter estimate	Standard error	*T*-statistic	DF	*p*
Face-selective	Subjective logit posterior of portrait gallery across all runs	0.048	0.056	0.863	184.000	0.389
	Inaccuracy penalty	0.001	0.056	0.021	184.000	0.983
	Slider displacement	0.009	0.056	0.160	184.000	0.873
Place-selective	Subjective logit posterior of landscape gallery across all runs	0.041	0.056	0.735	184.000	0.463
	Inaccuracy penalty	−0.003	0.056	−0.047	184.000	0.963
	Slider displacement	−0.015	0.056	−0.266	184.000	0.790

Supplementary Table 7. Fixed-effects parameter estimates for activation of face and place functional regions of interest (fROIs) by the logit prior of the gallery signaled by their preferred stimuli and logit likelihood conditional on the gallery signaled by their preferred stimuli. Corresponds to **Supplementary Figure 7b–c**. *DF*: Degrees of freedom (from Satterthwaite approximation[4]). *p*: *p*-value. Across 23 participants. The shading was included to aid in the visual separation of the rows for each fROI.

fROI	Regressor	Parameter estimate	Standard error	*T*-statistic	DF	*p*
Face-selective	Logit prior of portrait gallery across all runs	−0.011	0.061	−0.174	276.000	0.862
	Logit likelihood conditional on portrait gallery across all runs	0.170	0.061	2.783	276.000	0.006
	Inaccuracy penalty	−0.007	0.061	−0.118	276.000	0.906
	Slider displacement	0.019	0.061	0.318	276.000	0.751
Place-selective	Logit prior of landscape gallery across all runs	0.067	0.061	1.095	276.000	0.275
	Logit likelihood conditional on landscape gallery across all runs	−0.023	0.061	−0.372	276.000	0.710
	Inaccuracy penalty	−0.005	0.061	−0.082	276.000	0.935
	Slider displacement	−0.003	0.061	−0.052	276.000	0.958

Supplementary Table 8. Three-way ANOVA for effects of participant, contrast, and functional region of interest (fROI) on mean signal of the face and place fROIs, with focus on the subjective logit posterior.
Predictors:
"participant": identifier for each participant (23 total)
"contrast": (1) subjective logit posterior of portrait gallery only during portrait runs, (2) subjective logit posterior of landscape gallery only during landscape runs, (3) inaccuracy penalty, (4) slider displacement
"fROI": (1) face-selective fROI and (2) place-selective fROI

Predictor	Sum of Squares	DF	Mean Squares	*F*-statistic	*p*
participant	1.558	22	0.071	2.150	0.004
contrast	0.460	3	0.152	4.625	0.004
fROI	0.008	1	0.008	0.258	0.612
contrast*fROI	0.083	3	0.028	0.841	0.473
Error	5.072	154	0.033		
Total	7.178	183			

Supplementary Table 9. Three-way ANOVA for effects of participant, contrast, and functional region of interest (fROI) on mean signal of the face and place fROIs, with focus on logit prior and logit likelihood.
Predictors:
“participant”: identifier for each participant (23 total)
“contrast”: (1) logit prior of portrait gallery only during portrait runs, (2) logit prior of landscape gallery only during landscape runs, (3) logit likelihood conditional on portrait gallery only during portrait runs, (4) logit likelihood conditional on landscape gallery only during landscape gallery, (5) inaccuracy penalty, (6) slider displacement
“fROI”: (1) face-selective fROI and (2) place-selective fROI

Predictor	Sum of Squares	DF	Mean Squares	*F*-statistic	*p*
participant	1.665	22	0.076	2.015	0.006
contrast	0.621	5	0.124	3.307	0.007
fROI	0.022	1	0.022	0.584	0.445
contrast*fROI	0.179	5	0.036	0.954	0.447
Error	9.087	242	0.038		
Total	11.574	275			

Supplementary Table 10. Activation tracking subjective logit posterior by gallery category.

Contrast	Significant Cluster(s)?
Subjective logit posterior of portrait gallery	**Supplementary Figure 8**
Subjective logit posterior of landscape gallery	No

Supplementary Table 11. Activation tracking logit prior and logit likelihood by gallery category.

Contrast	Significant Cluster(s)?
Logit prior of portrait gallery	**Supplementary Figure 9**
Logit prior of landscape gallery	No
Logit likelihood conditional on portrait gallery	No
Logit likelihood conditional on landscape gallery	No

Supplementary References

1. Carpenter, R. H. S. & Williams, M. L. L. Neural computation of log likelihood in control of saccadic eye movements. *Nature* **377**, 59–62 (1995).
2. Roitman, J. D. & Shadlen, M. N. Response of Neurons in the Lateral Intraparietal Area during a Combined Visual Discrimination Reaction Time Task. *J. Neurosci.* **22**, 9475–9489 (2002).
3. Bechtold, B. Violin Plots for Matlab. (2016). doi:10.5281/zenodo.4559847
4. Luke, S. G. Evaluating significance in linear mixed-effects models in R. *Behav. Res. Methods* **49**, 1494–1502 (2017).
5. Eickhoff, S. B. *et al.* A new SPM toolbox for combining probabilistic cytoarchitectonic maps and functional imaging data. *Neuroimage* **25**, 1325–1335 (2005).
6. Eickhoff, S. B., Heim, S., Zilles, K. & Amunts, K. Testing anatomically specified hypotheses in functional imaging using cytoarchitectonic maps. *Neuroimage* **32**, 570–582 (2006).
7. Eickhoff, S. B. *et al.* Assignment of functional activations to probabilistic cytoarchitectonic areas revisited. *Neuroimage* **36**, 511–521 (2007).
8. Desikan, R. S. *et al.* An automated labeling system for subdividing the human cerebral cortex on MRI scans into gyral based regions of interest. *Neuroimage* **31**, 968–980 (2006).
9. Rolls, E. T., Huang, C. C., Lin, C. P., Feng, J. & Joliot, M. Automated anatomical labelling atlas 3. *Neuroimage* **206**, 116189 (2020).
10. Glasser, M. F. *et al.* A multi-modal parcellation of human cerebral cortex. *Nature* **536**, 171–178 (2016).
