# Supplementary material for: The parieto-occipital cortex is a candidate neural substrate for the human ability to approximate Bayesian inference
Source: Commun Biol. 2024 Feb 9;7:165. doi: 10.1038/s42003-024-05821-6 (PMC10858241; doi:10.1038/s42003-024-05821-6)
Supplement: Supplementary file 2 — Description of Additional Supplementary Files [file 42003_2024_5821_MOESM2_ESM.pdf]

## Description of Additional Supplementary Files

**File name:** Supplementary Data 1

**Description:** The list of trials in the scan session and their corresponding questioned gallery, prior probability, evidence strength, objective posterior probability, penalty, true hidden gallery, and sample picture.
