# Supplementary material for: The parieto-occipital cortex is a candidate neural substrate for the human ability to approximate Bayesian inference
Source: Commun Biol. 2024 Feb 9;7:165. doi: 10.1038/s42003-024-05821-6 (PMC10858241; doi:10.1038/s42003-024-05821-6)
Supplement: Supplementary file 4 — Reporting Summary [file 42003_2024_5821_MOESM4_ESM.pdf]

nature portfolio

Corresponding author(s): Nicholas M. Singletary
Jacqueline Gottlieb
Guillermo Horga

Last updated by author(s): Dec 26, 2023

Reporting Summary

Nature Portfolio wishes to improve the reproducibility of the work that we publish. This form provides structure for consistency and transparency in reporting. For further information on Nature Portfolio policies, see our Editorial Policies and the Editorial Policy Checklist.

Statistics

For all statistical analyses, confirm that the following items are present in the figure legend, table legend, main text, or Methods section.

n/a	Confirmed	
☐	☒	The exact sample size (*n*) for each experimental group/condition, given as a discrete number and unit of measurement
☐	☒	A statement on whether measurements were taken from distinct samples or whether the same sample was measured repeatedly
☐	☒	The statistical test(s) used AND whether they are one- or two-sided<br>*Only common tests should be described solely by name; describe more complex techniques in the Methods section.*
☐	☒	A description of all covariates tested
☐	☒	A description of any assumptions or corrections, such as tests of normality and adjustment for multiple comparisons
☐	☒	A full description of the statistical parameters including central tendency (e.g. means) or other basic estimates (e.g. regression coefficient) AND variation (e.g. standard deviation) or associated estimates of uncertainty (e.g. confidence intervals)
☐	☒	For null hypothesis testing, the test statistic (e.g. *F*, *t*, *r*) with confidence intervals, effect sizes, degrees of freedom and *P* value noted<br>*Give P values as exact values whenever suitable.*
☒	☐	For Bayesian analysis, information on the choice of priors and Markov chain Monte Carlo settings
☒	☐	For hierarchical and complex designs, identification of the appropriate level for tests and full reporting of outcomes
☐	☒	Estimates of effect sizes (e.g. Cohen's *d*, Pearson's *r*), indicating how they were calculated

Our web collection on statistics for biologists contains articles on many of the points above.

Software and code

Policy information about availability of computer code

Data collection	All tasks were written and presented to participants in MATLAB R2018 using extensions from Psychtoolbox.
Data analysis	Data were analyzed in MATLAB (versions R2018b, R2021a, and R2022a). fMRI data were analyzed with the following MATLAB extensions: SPM12 (Version 7487) and SnPM13.1.08. Custom code is available on Open Science Framework (https://osf.io/3vdut/?view_only=d89742283a84454681d59236c1e8b3b8).

For manuscripts utilizing custom algorithms or software that are central to the research but not yet described in published literature, software must be made available to editors and reviewers. We strongly encourage code deposition in a community repository (e.g. GitHub). See the Nature Portfolio guidelines for submitting code & software for further information.

Data

Policy information about availability of data

All manuscripts must include a data availability statement. This statement should provide the following information, where applicable:

- Accession codes, unique identifiers, or web links for publicly available datasets
- A description of any restrictions on data availability
- For clinical datasets or third party data, please ensure that the statement adheres to our policy

Experimental data are available at Open Science Framework (https://osf.io/3vdut/?view_only=d89742283a84454681d59236c1e8b3b8).

Research involving human participants, their data, or biological material

Policy information about studies with human participants or human data. See also policy information about sex, gender (identity/presentation), and sexual orientation and race, ethnicity and racism.

Reporting on sex and gender	The sample consisted of 23 participants (15 male, 8 female).
Reporting on race, ethnicity, or other socially relevant groupings	Participants were not categorized by race in this study.
Population characteristics	All participants were between 18 and 65 years of age.
Recruitment	We recruited participants through fliers posted on the Columbia University campus and through the recruitment system for the Columbia Business School Behavioral Research Lab. This pool consisted of Columbia University students, other Columbia affiliates, and affiliates of other universities in the New York Metropolitan Area, and they did not report any psychiatric or neurological disorders. Although participants varied by age (from 18 to 65), occupation, and socioeconomic status, participants were disproportionately young, U.S. American, and of higher socioeconomic status than the global average, potentially confounding application to the broader global population.
Ethics oversight	Columbia University Institutional Review Board

Note that full information on the approval of the study protocol must also be provided in the manuscript.

Field-specific reporting

Please select the one below that is the best fit for your research. If you are not sure, read the appropriate sections before making your selection.

☒ Life sciences ☐ Behavioural & social sciences ☐ Ecological, evolutionary & environmental sciences

For a reference copy of the document with all sections, see nature.com/documents/nr-reporting-summary-flat.pdf

Life sciences study design

All studies must disclose on these points even when the disclosure is negative.

Sample size	We chose a sample size comparable to that of other recent human cognitive neuroscience studies.
Data exclusions	We initially recruited 38 participants (excluding 6 participants who withdrew from the study). Fourteen of these participants were excluded because their responses during a prescan session reflected disengagement or lack of comprehension according to predetermined performance-based exclusion criteria. Another participant was excluded because excessive head motion during scanning rendered their fMRI data unusable.
Replication	Results were replicated in 23 participants
Randomization	Covariates were controlled for using regression.
Blinding	Blinding was not relevant because participants were not assigned to experimental groups.

Reporting for specific materials, systems and methods

We require information from authors about some types of materials, experimental systems and methods used in many studies. Here, indicate whether each material, system or method listed is relevant to your study. If you are not sure if a list item applies to your research, read the appropriate section before selecting a response.

Materials & experimental systems

n/a	Involved in the study	
☒	☐	Antibodies
☒	☐	Eukaryotic cell lines
☒	☐	Palaeontology and archaeology
☒	☐	Animals and other organisms
☒	☐	Clinical data
☒	☐	Dual use research of concern
☒	☐	Plants

Methods

n/a	Involved in the study	
☒	☐	ChIP-seq
☒	☐	Flow cytometry
☐	☒	MRI-based neuroimaging

Plants

Seed stocks

Report on the source of all seed stocks or other plant material used. If applicable, state the seed stock centre and catalogue number. If plant specimens were collected from the field, describe the collection location, date and sampling procedures.

Novel plant genotypes

Describe the methods by which all novel plant genotypes were produced. This includes those generated by transgenic approaches, gene editing, chemical/radiation-based mutagenesis and hybridization. For transgenic lines, describe the transformation method, the number of independent lines analyzed and the generation upon which experiments were performed. For gene-edited lines, describe the editor used, the endogenous sequence targeted for editing, the targeting guide RNA sequence (if applicable) and how the editor was applied.

Authentication

Describe any authentication procedures for each seed stock used or novel genotype generated. Describe any experiments used to assess the effect of a mutation and, where applicable, how potential secondary effects (e.g. second site T-DNA insertions, mosiacism, off-target gene editing) were examined.

Magnetic resonance imaging

Experimental design

Design type

Task; event-related

Design specifications

There were 130 trials per participant. Each trial lasted 18 s.

Behavioral performance measures

Participants submitted their responses (subjective posterior probability) by using a trackball to move a slider on the screen and clicking a button on the trackball to submit their response. To determine whether participants were performing the task as expected, we measured the correlation between subjective and objective posterior probability on catch trials, and we tested for significant difference in subjective posterior probability on trials with the highest (> 0.9) and lowest (< 0.1) objective posterior probability. Reaction times (time to submit) were also recorded.

Acquisition

Imaging type(s)

Structural and functional

Field strength

3T

Sequence & imaging parameters

Pulse sequence: spin echo, Imaging type: EPI, FOV: 230 mm, Slice thickness: 2.40 mm, Orientation: transversal, TE: 30.00 ms, TR: 1000 ms, Flip angle: 52 deg

Area of acquisition

Whole-brain

Diffusion MRI ☐ Used ☒ Not used

Preprocessing

Preprocessing software

Preprocessing was performed using the fMRIPrep pipeline, Version 1.5.0rc1 (RRID:SCR_016216). fMRIPrep uses a combination of tools from well-known software packages, including FSL, ANTs, FreeSurfer, and AFNI, and is based on Nipype 1.2.0 (RRID:SCR_002502). For more details of the pipeline, see the section corresponding to workflows in fMRIPrep's documentation at (https://fmriprep.org/en/latest/workflows.html).

The T1-weighted (T1w) image was corrected for intensity nonuniformity with N4BiasFieldCorrection, distributed with ANTs 2.2.0 (RRID:SCR_004757). The T1w image was then skull-stripped with a Nipype implementation of the antsBrainExtraction.sh workflow (from ANTs), using OASIS30ANTs as target template. Brain tissue segmentation of cerebrospinal fluid, white matter, and gray matter was performed on the brain-extracted T1w using fast (FSL 5.0.9, RRID:SCR_002823). Volume-based spatial normalization to Montreal Neurological Institute (MNI) space (MNI152NLin2009cAsym) was performed through nonlinear registration with antsRegistration (ANTs 2.2.0) (RRID:SCR_008796).

A skull-stripped susceptibility distortion–corrected BOLD reference was generated using a custom methodology of fMRIPrep. The BOLD reference was co-registered to the T1w reference using bbregister (FreeSurfer), which implements boundary-based registration using six degrees of freedom. Head-motion parameters (x, y, z, pitch, roll, and yaw) with respect to the BOLD reference were estimated before spatiotemporal filtering using mcflirt (FSL 5.0.9). BOLD runs were slice-time corrected using 3dTshift from AFNI 20160207 (RRID:SCR_005927).

Normalization

Volume-based spatial normalization to Montreal Neurological Institute (MNI) space (MNI152NLin2009cAsym) was performed through nonlinear registration with antsRegistration (ANTs 2.2.0) (RRID:SCR_008796).

Normalization template

MNI152NLin2009cAsym

Noise and artifact removal

Head-motion parameters (x, y, z, pitch, roll, and yaw) with respect to the BOLD reference were estimated before spatiotemporal filtering using mcflirt (FSL 5.0.9). BOLD runs were slice-time corrected using 3dTshift from AFNI 2016020791

	(RRID:SCR_005927).
Volume censoring	No volumes were censored.

Statistical modeling & inference

Model type and settings	First-level (participant-level) models were analyzed using a mass-univariate approach. Second-level (group-level) maps were carried out on the contrast maps generated by the first-level analysis. To produce second-level whole-brain maps, we used applied a cluster-wise correction for multiple comparison using non-parametric permutation tests.
Effect(s) tested	We measured the effects of subjective logit posterior, objective logit posterior, logit prior, and logit likelihood. Parametric designs were used.

Specify type of analysis: ☐ Whole brain ☐ ROI-based ☒ Both

Anatomical location(s)	The anatomical location of the parieto-occipital ROI was determined from a significant cluster that exhibited effects of subjective logit posterior at the second level. Anatomical locations of the face- and place-selective regions were determined by a separate face–place localizer.

Statistic type for inference (See Eklund et al. 2016)	Cluster wise inference. Cluster-defining p-value height threshold: 0.001. Family-wise-error-rate threshold for cluster significance 0.05
Correction	FWE, permutation test

Models & analysis

n/a	Involved in the study
☒	☐ Functional and/or effective connectivity
☒	☐ Graph analysis
☒	☐ Multivariate modeling or predictive analysis
